# Mining whole genome sequence data to efficiently attribute individuals to source populations

**DOI:** 10.1038/s41598-020-68740-6

**Published:** 2020-07-22

**Authors:** Francisco J. Pérez-Reche, Ovidiu Rotariu, Bruno S. Lopes, Ken J. Forbes, Norval J. C. Strachan

**Affiliations:** 1 Institute of Complex Systems and Mathematical Biology, SUPA, School of Natural and Computing Sciences, University of Aberdeen, Aberdeen, AB24 3UE Scotland UK; 2 School of Biological Sciences, University of Aberdeen, Aberdeen, AB24 3UU Scotland UK; 3 School of Medicine, Medical Sciences and Dentistry, University of Aberdeen, Foresterhill, Aberdeen, AB25 2ZD Scotland UK

**Keywords:** Population genetics, Evolutionary genetics, Bacterial evolution, Scientific data

## Abstract

Whole genome sequence (WGS) data could transform our ability to attribute individuals to source populations. However, methods that efficiently mine these data are yet to be developed. We present a minimal multilocus distance (MMD) method which rapidly deals with these large data sets as well as methods for optimally selecting loci. This was applied on WGS data to determine the source of human campylobacteriosis, the geographical origin of diverse biological species including humans and proteomic data to classify breast cancer tumours. The MMD method provides a highly accurate attribution which is computationally efficient for extended genotypes. These methods are generic, easy to implement for WGS and proteomic data and have wide application.

## Introduction

**Figure 1:**
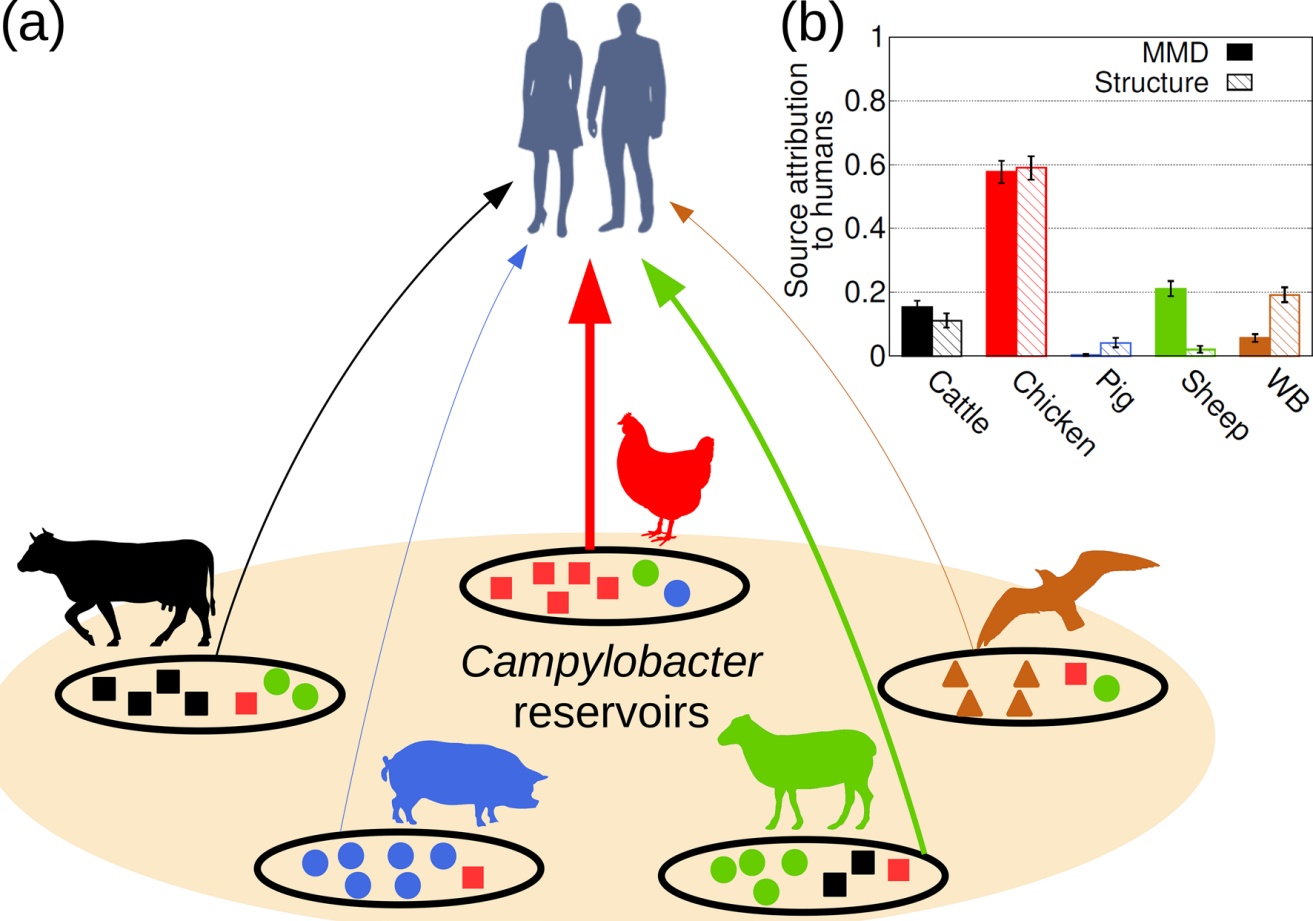
Source attribution. The general aim of source attribution (or assignment tests) is to determine the probability $p_{u,s}$ that an individual of unknown origin, *u*, originates from a certain source, *s*. (**a**) Provides the set of source populations considered in this study for *Campylobacter*: cattle, chicken, pigs, sheep and wild birds. The genetic profile of a source population *s* is represented by the genotypes of a set of $I_s$ individuals sampled from the source. Different symbols within sources schematically depict different genotypes. The genotype is determined from a set of genetic markers (loci) that depend on the typing method. (**b)** Provides the probability $p_s$ that any of the 500 human *Campylobacter* isolates are attributed to a source *s*. Results are shown for both MMD (solid bars) and STRUCTURE (hatched bars) methods, based on 25,937 cgSNP genotypes. All the silhouettes are recoloured versions of publicly available silhouettes: Cattle from https://pixabay.com/service/license/. Chicken, pig and sheep by Karen Arnold under CC0 Public Domain license, https://creativecommons.org/publicdomain/zero/1.0/. Wild bird by Andreas Plank licensed under the Creative Commons Attribution-Share Alike 3.0 Unported, https://creativecommons.org/licenses/by-sa/3.0/deed.en. Humans by Mia Irawati, https://www.kindpng.com/imgv/xoRmR_30-people-silhouettes-clip-arts-people-silhouette-png/.

Attributing or assigning individuals to a source population is important within many disciplines including ecology, anthropology, infectious diseases and forensics^1,2^. For instance, assignment tests have been applied to identify the origin of individuals in ecosystems^3–7^, infectious diseases^8–19^, animals used for trade^5^ or the geographical origin of humans^20–22^ or plants^23^. A common strategy to attribute individuals to populations consists in comparing the genotype of the individual with the genetic profiles of defined source populations (e.g. the infectious disease example depicted in Fig. 1). The genotype usually comprises a set of genetic markers selected to highlight the differences between individuals (Fig. 1). For instance, highly variable genetic markers such as microsatellites^3–7^ or genes^8,11–19,24^ and more recently single nucleotide polymorphisms (SNPs)^25^ have been used for source attribution. The question is to decide which approach is most appropriate for the particular problem in terms of computation time and assignment accuracy.

With the advent of next-generation sequencing technology, whole genome sequences are becoming available across all the 6 kingdoms of life ranging in size from for example viruses (kBases) to humans (3.2 GBases)^26–30^. In principle, this should enable discovery of large numbers of markers (e.g. SNPs) which have the potential to achieve unprecedented source attribution accuracy^31–36^. The challenge lies in efficiently mining large data sets for source attribution. Existing source attribution methods (e.g. STRUCTURE^37,38^ that has been widely applied in population genetics) operate on relatively short genotypes consisting of a few to tens or hundreds of loci. However, their computation time increases at least linearly with the number of loci and using extended genotypes (e.g. $> {1{,}000} \, \hbox {loci}$) is impractical. This is a particularly important drawback in situations where rapid source attribution is crucial (e.g. for infectious diseases). Source attribution based on extended genotypes therefore requires developing more efficient methods.

The limited effort to optimise source attribution algorithms to use extended genotypes contrasts with the effort made to address another important challenge in population structure, namely the use of extended genotypes to clustering individuals into groups. For instance, FRAPPE^39^, ADMIXTURE^40^, fastStructure^41^, fineStructure^42^, sNMF^43^, snapclust^44^, principal components analysis (PCA)^45^ or Discriminant analysis of principal components (DAPC)^46^ are well-known methods that can identify clusters using extended genotypes. In the language of machine learning^47^, these programs use unsupervised learning algorithms to infer clusters in the data without using any prior information about the characteristics of such clusters. Hence, such algorithms are not suitable for source attribution which requires supervised learning algorithms to classify individuals to a set of predefined sources whose characteristics are defined in terms of genotypes of known origin (i.e. in terms of a training set). ADMIXTURE was originally proposed as a method for unsupervised model-based estimation of ancestry of unrelated individuals^40^. This is the most widely used version of ADMIXTURE but, in fact, it was extended^48^ for supervised learning in such a way that it can use prior knowledge on the population of origin of some individuals to infer the ancestry of other individuals. The supervised learning version of ADMIXTURE, however, was not designed to estimate the probability that individuals were sampled from a certain source, i.e., it was not designed to attribute individuals to sources but rather to infer their ancestry. In spite of that, one would expect some relationship between ancestry and source of individuals and it makes sense to explore the capability of ADMIXTURE as an attribution method (with applicability restricted to datasets consisting of SNP genotypes). GLOBETROTTER, another package to infer the ancestry of individuals, also has potential as a method for source attribution with extended SNP datasets^49^.

Besides developing efficient methods for source attribution, selection of loci with high discriminatory power can also help deal with the computational challenge posed by extended genotypes. Several methods have been proposed to rank markers according to their importance for source attribution based on the intuitive idea that highly polymorphic markers should allow for higher genetic differentiation^50^. This can be achieved by measuring the importance of loci with diversity indices (e.g. expected heterozygosity, fixation index $F_{ST}$ or informativeness^5,7,21,51,52^). Other approaches propose focusing on the joint performance of sets of loci rather than considering performance of loci individually^53–55^. One would expect these approaches to be more appropriate than diversity-based methods when dealing with correlated markers (i.e. when linkage disequilibrium is important^56^). However, they are computationally intensive and impractical to deal with extended genotypes and do not always improve on diversity-based methods^7^.

Here, we address two of the challenges posed by extended genotypes for source attribution. First, we propose a fast method for source attribution which can deal with genotypes comprising thousands of loci with minimal computational effort. Second, we propose the use of information theory^57^ for the optimal selection of markers from extended genotypes. We demonstrate this through several examples. The first is in the field of infectious diseases and involves *Campylobacter*, the largest cause of human bacterial gastroenteritis in the developed world^58,59^. Here we attribute human cases to source reservoirs (e.g. chicken, cattle, sheep etc.). The second is in the area of human evolution and involves attributing humans to 7 reference regions (e.g. Africa, Europe, etc.) or 53 populations (e.g. Bedouin, Maya, etc.)^22,60,61^. The third example studies attribution of the giant Californian sea cucumber (*Parastichopus californicus*) to north/south subregions within the northeastern Pacific coastal region^62^. In the fourth example, we assign breast cancer tumours to three different subtypes (ERPR, Her2 and TN)^63^. The first three examples use genomic data and the breast cancer example uses proteomic data. The performance of our method for source attribution is compared to the current state of the art method STRUCTURE^37,38^. For extended human genotypes which are too computationally intensive for STRUCTURE, a comparison is made with the supervised learning ADMIXTURE method^48^ by assuming that the probability of attribution to a source can be identified using the ancestry coefficient corresponding to such a source.

## Results

### Source attribution with the MMD method

**Figure 2:**
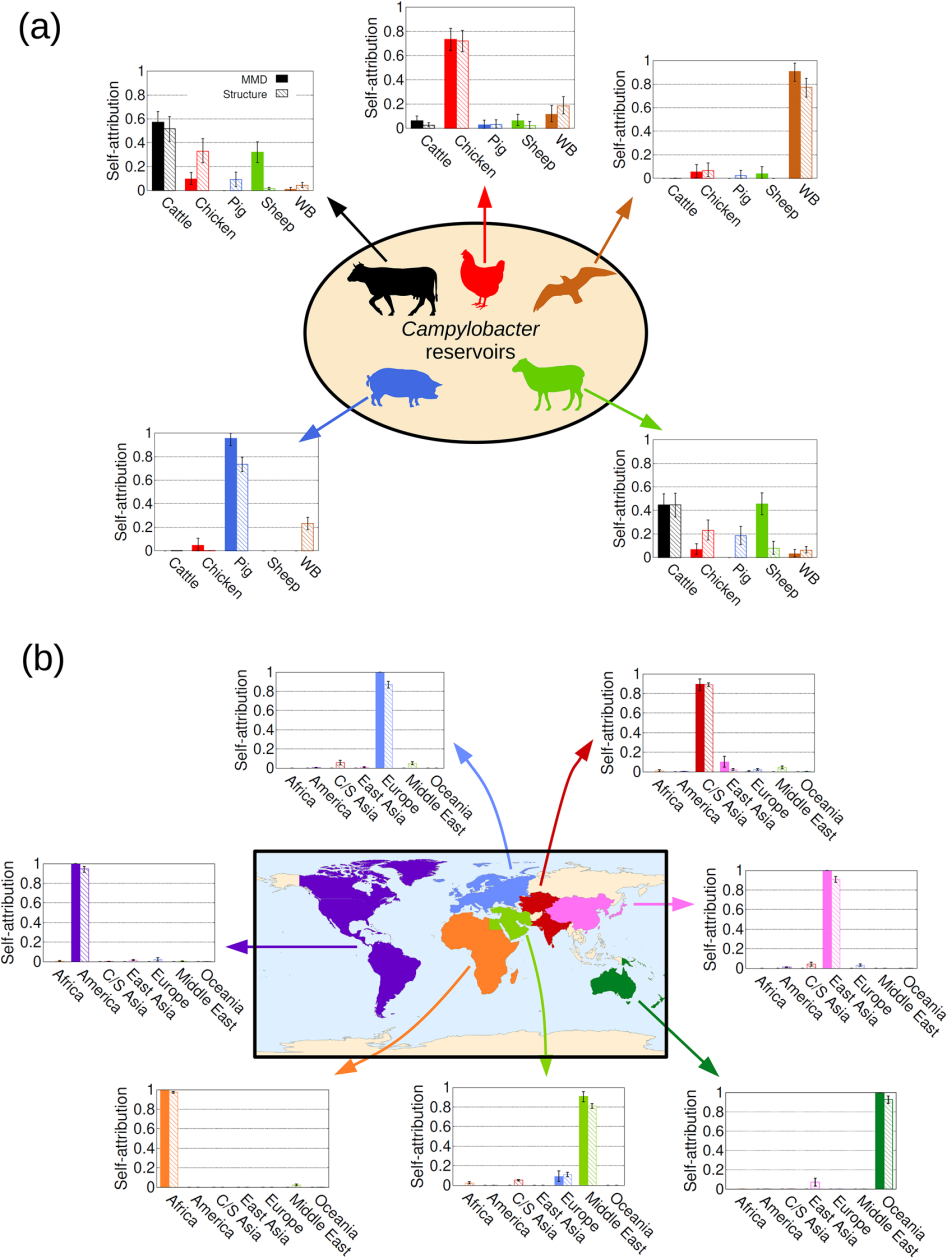
Self-attribution to test the accuracy of the source attribution methods. Self-attribution is a cross-validation strategy that involves removing individuals from the source populations and estimating the probability that they are correctly attributed to their source based on the remainder. Here $50\%$ are removed. The bar charts provide the probability distribution $p_s$ for (**a**) *Campylobacter* (genotypes described by 25,938 cgSNPs) and (**b**) humans (659,276 SNPs) comprising 5 and 7 source populations respectively. Bars in (**a**) show results obtained using the MMD method (solid bars) and STRUCTURE (hatched bars). Bars in (**b**) results obtained using the MMD method (solid bars) and ADMIXTURE (hatched bars). Perfect self-attribution would result in $100\%$ assignment to the appropriate source population. The total self-attribution accuracy when combining the results across all the source populations was, respectively, $73\%$ and $56\%$ for MMD and STRUCTURE in the *Campylobacter* population example. For the human population example, it was $97\%$ and $71\%$ for MMD and ADMIXTURE, respectively. All the silhouettes shown in (**a**) are recoloured versions of publicly available silhouettes: Cattle from https://pixabay.com/service/license/. Chicken, pig and sheep by Karen Arnold under CC0 Public Domain license, https://creativecommons.org/publicdomain/zero/1.0/. Wild bird by Andreas Plank licensed under the Creative Commons Attribution-Share Alike 3.0 Unported, https://creativecommons.org/licenses/by-sa/3.0/deed.en.

We propose the Minimal Multilocus Distance (MMD) method to estimate the probability $p_{u,s}$ that an individual *u* is attributed to a population source *s* based on the similarity between the genotype of the individual to be attributed and genotypes from the sources. The similarity between pairs of genotypes is quantified by the Hamming distance which simply gives the number of loci at which the genotypes differ^64^. The smaller the distance between genotypes, the larger the probability that they originate from the same source (see Methods). To test the accuracy of the MMD method, we studied self-attribution, a cross-validation method^65,66^ which consists in removing individuals from the source population and estimating the probability that they are correctly attributed to their source based on the remainder^5,12,13,51^ (Fig. 2). Within the context of machine learning, splitting the data into training and test sets is a general procedure to train and test the accuracy of classifiers^47^.

The source attribution results corresponding to a set of $I_u$ individuals (e.g. $I_u=500$ *Campylobacter* isolates from humans) are summarised by the probability distribution $p_s$ that any of the individuals is attributed to source *s* (see an example in Fig. 1b and more details in Methods). Self-attribution results are summarised by a similar probability distribution, assuming that the individuals that were removed from a population represent a set of $I_u$ individuals of unknown origin (see Fig. 2). In the following, we describe the results obtained for the *Campylobacter* and human examples. Self-attribution results for *P. californicus* genotypes and breast cancer proteomic data are described in Additional files 4 and 5, respectively.

**Figure 3:**
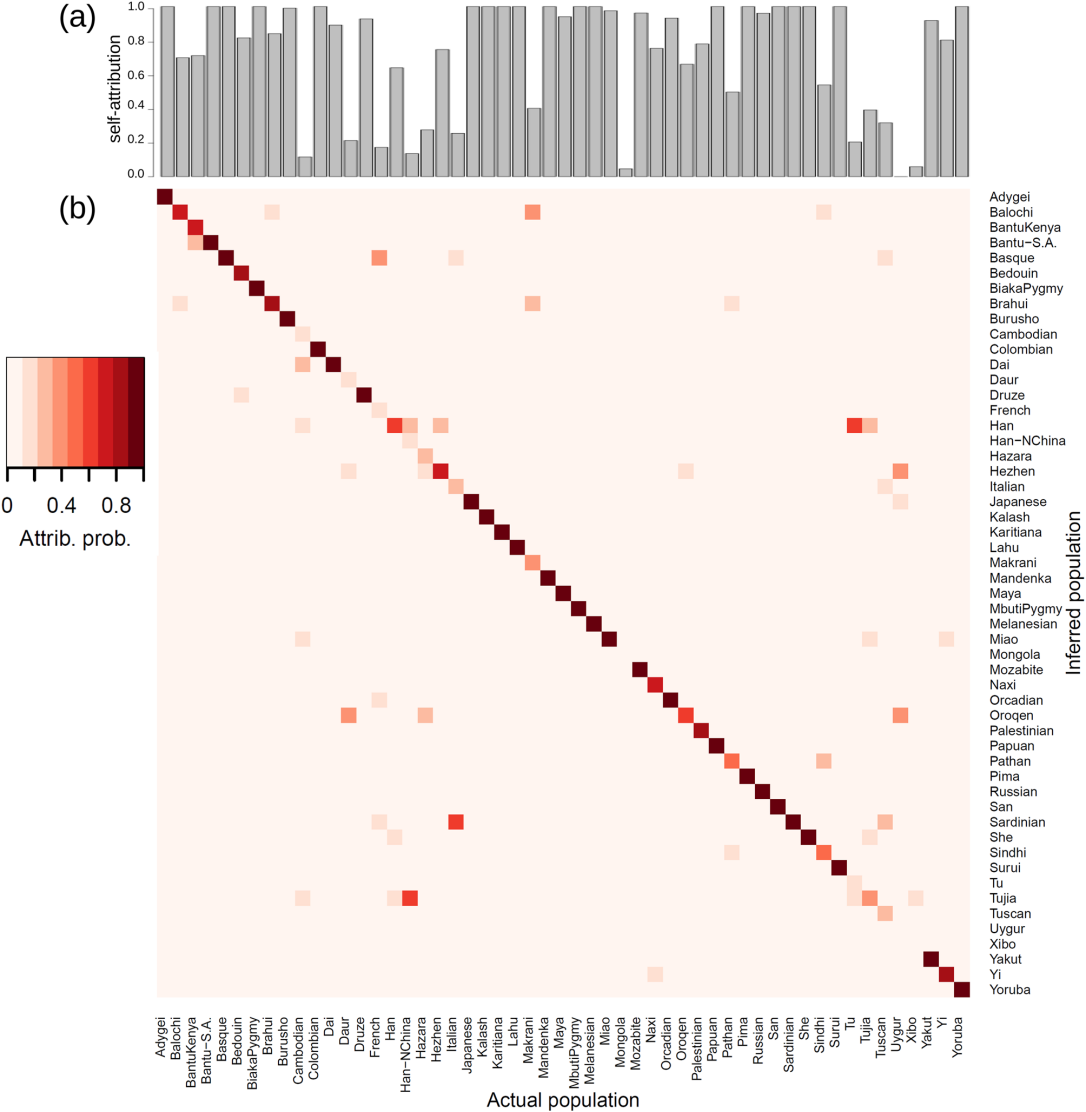
Self-attribution of humans to 53 sampling populations with MMD. Self-attribution for a given population was performed by randomly removing $50\%$ of the individuals from that population and then attributing them to the populations characterized by the remaining individuals. (**a**) Probability of correct self-attribution for individuals selected from each population. (**b**) Attribution probability $p_{u,s}$ of removed individuals, *u*, to each of the populations, *s*. Darker colours correspond to higher probability, see the colour legend. The horizontal axis gives the population from which individuals were sampled and the vertical axis gives the inferred attribution probability to each population.

#### Campylobacter

Self-attribution was carried out for isolates from food and animal sources by removing $50\%$ of the isolates for blind attribution (see Fig. 2a and Additional file 3: Table S6). Human clinical isolates are not considered for self-attribution since their source is unknown. The MMD method correctly assigned most isolates ($>70\%$) from pig, chicken and wild bird based on 25,937 core genome SNP (cgSNP) genotypes. Self-attribution of *Campylobacter* isolates from cattle and sheep is less precise (58% and 45%). Wrongly self-attributed cattle isolates are mostly assigned to sheep and chicken sources, whilst sheep isolates tend to be erroneously attributed to cattle and chicken sources. When combining the self-attribution results across all source populations, an overall attribution accuracy of 73% was obtained.

**Figure 4:**
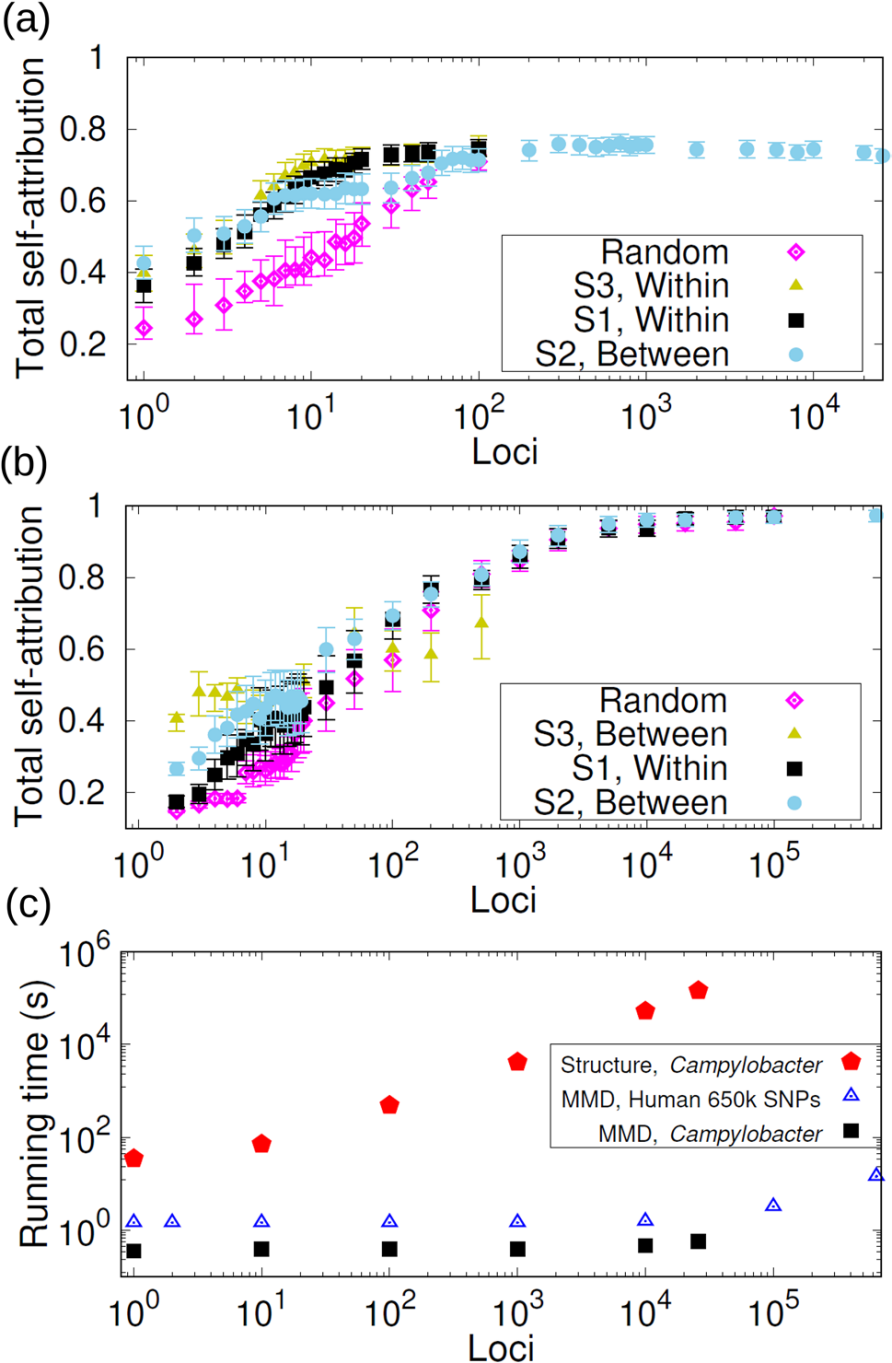
Selection of markers and computational times. (**a**) Total self-attribution probability $p^{\text {sa}}$ that any *Campylobacter* isolate from food reservoirs is correctly attributed to its source. The probability is plotted as a function of the number of cgSNPs selected at random and with strategies S1 (loci ranked in decreasing within-source diversity), S2 (loci ranked in decreasing between-source diversity) and S3 (reordering the loci ranking corresponding to S1 to reduce loci redundancy). (**b**) Similar representation for the total self-attribution probability of human individuals based on 659,276 SNPs. Strategy S3 reorders the loci ranking corresponding to S2 to reduce redundancy. (**c**) Squares and pentagons show the computational time required for MMD and STRUCTURE, respectively, to assign a *Campylobacter* genotype with number of cgSNPs ranging between 1 and 25,937. Triangles show the time required for MMD to assign a human genotype with number of SNPs ranging between 1 to 659,276.

Source attribution was then carried out to predict the origin of the *Campylobacter* that resulted in human infection. As shown in Fig. 1, MMD estimated that most cases ($61\%$) were associated with chicken whilst wild birds and pigs were relatively unimportant ($<8\%$ for both sources). This is in line with a number of previous source attribution studies for human campylobacteriosis^11–19^.

*Human*. MMD self-attribution accuracy, based on removal of $50\%$ of individuals genotyped at 659,276 SNPs^60^, was $100\%$ accurate for all regions except for C/S Asia ($90\%$) and Middle-East ($91\%$). An overall self-attribution accuracy of $97\%$ was obtained in this case (see Fig. 2b and Additional file 3: Table S9).

Self-attribution based on 659,276 SNP genotypes was also studied at the level of the 53 populations available in the dataset^60^. In this case, an overall self-attribution accuracy of $73\%$ was obtained. More explicitly, self-attribution accuracy was $>64\%$ for 38 populations (see Fig. 3). Accuracy was poor for several populations from C/S Asia, E. Asia and Europe. For instance, individuals from the Uygur population (C/S Asia) were attributed to three populations in East Asia: Oroqen ($40\%$), Hezhen ($40\%$) and Japanese ($20\%$). The attribution of individuals from some populations in East Asia (e.g. Mongola, Xibo, Cambodian, Han-NChina) was spread over other populations from East Asia. For the European region, individuals from French, Italian and Tuscan populations were often attributed to other geographically close populations. For instance, $17\%$ of French individuals were correctly self-attributed, $39\%$ were attributed to the Basque population, $17\%$ to Orcadian, $13\%$ to Sardinian, $10\%$ to Italian and $4\%$ to Tuscan.

### Comparison with structure and admixture

The MMD method was compared with the current state of the art method STRUCTURE^37,38^ both in terms of attribution accuracy and computational speed. We used the *Campylobacter* and human genotypes for the comparison. Assuming that each source corresponds to a genetically-distinct population, STRUCTURE uses Bayesian inference to estimate the source attribution probability $p_{u,s}$ (see details on the implementation of STRUCTURE in Methods).

#### Campylobacter

Self-attribution tests for *Campylobacter* genotypes suggests that the probability of correct assignment calculated with the MMD method is higher than that obtained with STRUCTURE for all the reservoirs (overall $73\%$ for MMD and $57\%$ for STRUCTURE, see Fig. 2a and Additional file 3: Table S6). Both MMD and STRUCTURE have poorer self-attribution accuracy for cattle and sheep; the largest difference between MMD and STRUCTURE is observed for sheep isolates which are poorly attributed by STRUCTURE. In terms of source attribution of human *Campylobacter* isolates, both methods gave similar results with chicken being the most and pigs being the least important (Fig. 1b).

#### Human

A comparison of STRUCTURE and the MMD method based on extended 659,276 SNP human genotypes is not practical due to the long running time for STRUCTURE. In order to compare with STRUCTURE for humans, we considered smaller genotypes comprising 645 microsatellites^22^ and 2,810 SNPs^61^. For the microsatellite genotypes, MMD and STRUCTURE give similar overall self-attribution ($87\%$ compared with $84\%$, see Additional file 2: Fig. S1 and Additional file 3: Table S7). Both MMD and STRUCTURE find it more difficult to differentiate between the European and Middle Eastern populations (Additional file 2: Fig. S1), due to a proportion of individuals in the European region being classified as Middle Eastern and vice-versa. When using the 2,810 SNP data set, STRUCTURE performed better with an overall attribution of $91\%$ compared with $79\%$ for MMD. The largest difference between MMD and STRUCTURE is observed for individuals from C/S Asia which are poorly attributed by the MMD method (Additional file 2: Fig. S1 and Additional file 3: Table S8).

In order to compare the MMD method with existing methods for extended human genotypes comprising 659,276 SNPs, we performed supervised analyses of ancestry using ADMIXTURE^48^ (see Methods for a more detailed description of ADMIXTURE implementation). The overall self-attribution accuracy achieved with ADMIXTURE is quite high ($90\%$) but it is lower than for MMD ($97\%$), see Fig. 2b and Additional file 3: Table S9. In fact, self-attribution based on ADMIXTURE is less accurate than that obtained with MMD for all the regions. The largest differences between the self-attribution accuracy of the two methods were obtained for European individuals ($100\%$ with MMD and $87\%$ with ADMIXTURE). This is mainly due to a significant contribution of C/S Asia and Middle East to the ancestry of Adygei individuals (see Additional file 2: Fig. S3). The self-attribution differences between the two methods are $\le 10\%$ for all regions except Europe. In particular, the smallest difference was observed for individuals from C/S Asia ($90\%$ with MMD and $89\%$ with ADMIXTURE). In this case, MMD predicts a small probability of attribution to East Asia. ADMIXTURE predicts small probabilities of attribution to Middle East, Europe, East Asia and Africa (see Fig. 2b, Additional file 2: Fig. S3 and Additional file 3: Table S9).

Our application of ADMIXTURE gives attribution accuracies below $90\%$ for individuals from C/S Asia ($89\%$ correctly attributed), Europe ($87\%$ correctly attributed) and Middle East ($81\%$). For several of the individuals selected from Europe, ADMIXTURE predicts a rather mixed ancestry from several regions other than Europe (see Additional file 2: Fig. S3 and Additional file 3: Table S9). For instance, Middle Eastern ancestry is inferred for some Italian, Sardinian, Tuscan and Adygei individuals. C/S Asian ancestry is predicted for Adygei, Russian and French individuals. ADMIXTURE also predicts a significantly mixed ancestry for Middle East individuals. In this case, C/S Asia and European ancestry is predicted for Druze, Palestinian and, to a lower extent, Bedouin individuals. African ancestry is predicted for all Mozabite and some Bedouin and Palestinian individuals.

#### Computational time

The computational time for MMD is much shorter than STRUCTURE. Figure 4c shows a comparison of runtimes for self-attribution of *Campylobacter* isolates as a function of the number of SNP loci describing the genotypes. The MMD is between 100 and $10^5$ times faster than STRUCTURE for every run from 1 to $\sim 2 \times 10^4$ SNPs. Since the running time of MMD increases slowly with the number of loci compared to that of STRUCTURE, the efficiency of MMD improves relative to that of STRUCTURE for extended genotypes. For instance, STRUCTURE takes $\sim 40$ h to assign a 25,937 cgSNP genotype whereas MMD completes the task in $\sim 0.57$ s (MMD implementation in R^67^, Processor: Intel Core i7-3770 3.40 GHz).

The MMD method is around twice as fast as ADMIXTURE when considering the 659,276 SNP dataset. More explicitly, MMD takes $\sim 15$ s to assign an individual whereas ADMIXTURE takes $\sim 38$ s to infer the ancestry of one individual (times based on an Intel Core i7-3770 3.40 GHz processor for both algorithms).

For a given number of loci, the running time of MMD for attribution of a *Campylobacter* isolate is systematically smaller than the running time for attribution of a human individual from the 659,276 SNP dataset (see Fig. 4c). This is mostly due to the longer initial start-up time needed to deal with the human dataset which is significantly longer than that of *Campylobacter*. The running time remains essentially constant for runs with less than $10^5$ loci since the start-up time dominates over the MMD algorithm computational time which increases with the Hamming distance itself, not the number of loci (see Methods).

### Selecting informative markers for source attribution

In general, one would expect that the assignment power of a set of *n* markers will be subject to the following two conditions: (C1) Markers should allow us to capture the genetic differences between sources, i.e. the allele distribution of selected loci should significantly differ between sources. (C2) The *n* markers should contain complementary genetic information relevant to the source attribution process and ideally no redundant information. For example, the discriminatory power achieved with a set of markers will not increase significantly if a marker is added which brings redundant information compared to those that were already selected. Information theory offers a natural framework to account for allele diversity (relevant to C1) and loci redundancy (relevant to C2). Within this framework, allele diversity was quantified by the Shannon entropy and loci redundancy by the mutual information between pairs of loci (see Methods).

Inspired by these conditions, we propose *three strategies* to build sets of markers with high assignment power. Strategy S1 ranks loci in order of decreasing allele diversity *within* sources. Strategy S2 ranks loci in order of decreasing allele diversity *between* sources. Strategy S3 uses a greedy procedure^54^ that rearranges the loci obtained with S1 and S2 to reduce the redundancy. More explicitly, the list of selected loci in S3 is built by adding loci one by one making sure that the locus selected at the *n*th step brings the smallest possible redundancy compared to the $n-1$ previously selected loci. See the Methods section for an explicit definition of the redundancy $R_n$ of the *n*th locus. Due to its greedy nature, strategy S3 is computationally more demanding than S1 and S2 and was applied to a limited number of loci to fine tune the selection of S1 or S2 (we only present the results of applying S3 to reorder the ranking given by the better performing strategy S1 or S2). The self-attribution performance of the three strategies as well as random selection of loci is illustrated in Fig. 4a for *Campylobacter*. As the number of selected SNPs increases, all of the targeted strategies (S1–S3) saturated more quickly than random selection. The strategy that requires the fewest SNPs (approximately 10) to obtain optimal self-attribution is S1.

This is repeated for the human population examples. For the human 659,276 SNPs dataset, S2 does better than S1 for small numbers of loci but the difference becomes undistinguishable for $n > 100$ SNPs (see Fig. 4b). Strategy S3 brings some improvement over S2 for selections of $n <10$ loci but does worse when selecting more loci. All tested strategies lead to saturation of the self-attribution accuracy when $\sim 10^4$ SNPs are selected. For the microsatellite data set, strategy S2 also does better than S1 and strategy S3 does not improve on S2. Irrespective of the loci selection strategy, no sign of saturation of the total self-attribution is observed for the available loci (Additional file 2: Fig. S4). For the human 2,810 SNP genotypes, S2 does significantly better than S1 and S3 fractionally improves on S2 for selections of less than $\sim 10$ loci (Additional file 2: Fig. S5). However, the fraction of correctly self-attributed individuals increases slowly with the number of selected loci and using S3 does not represent a real advantage.

## Discussion

The source attribution problems studied here belong to a wider class of population structure challenges that also include classifying individuals in clusters of common features without assuming the population structure a priori. Significant effort has been made to optimise clustering algorithms to address the later challenge^39–46,48,68^. In contrast, optimisation of source attribution algorithms to use extended genotypes has received limited attention. The MMD method proposed here aims at filling this crucial gap.

Self-attribution tests were used to estimate the source attribution accuracy of MMD, STRUCTURE and supervised ADMIXTURE. This type of test is widely used to test the accuracy of supervised classifiers^47^ using data from known sources. The ultimate aim of source attribution, however, is to determine the source of individuals whose origin is genuinely unknown. This is the case for human *Campylobacter* isolates that we attributed to animal sources using MMD and STRUCTURE. The fact that the self-attribution accuracy of both MMD and STRUCTURE is reasonably high gives confidence on the accuracy of source attribution of human isolates. In addition to source attribution, in the future it would be interesting to test the accuracy of these methods for source attribution of human *Campylobacter* isolates from outbreaks with known source. Genomic data from *Campylobacter* outbreaks is, however, limited and this might prevent a detailed analysis at the moment.

The self-attribution accuracy of the MMD method is better than that of STRUCTURE for the *Campylobacter* example using SNPs ($73\%$ vs. $57\%$), approximately the same for the human origin from 7 geographical regions using microsatellites ($87\%$ v $84\%$) and slightly poorer than the human origin example using 2,810 SNPs ($79\%$ vs. $91\%$). These results indicate its potential as an alternative to STRUCTURE. The MMD self-attribution accuracy of humans to 7 geographical regions increased to $97\%$ when using 659,276 SNPs. This is better than the $90\%$ self-attribution accuracy achieved by using the ancestry inferred by ADMIXTURE for the same dataset. A comparison with STRUCTURE was impractical for this dataset. The MMD method also gave a high self-attribution accuracy ($73\%$) when using 659,276 SNPs to assign humans to 53 populations.

Self-attribution of *Campylobacter* isolates from cattle and sheep reservoirs is poor compared to other reservoirs for both MMD and STRUCTURE methods. Similar trends have been reported in previous studies on *Campylobacter* self-attribution (see, e.g.^8^). This is likely due to the similarity of niche in cattle and sheep as both are ruminants. Also, geographical proximity offers frequent opportunities of transmission between the populations and this would explain the high genetic proximity between *Campylobacter* isolates from the cattle and sheep reservoirs (see Additional file 3: Table S1 where the allele-frequency divergence^69^ has been used as a measure of the genetic differentiation between sources).

Self-attribution of humans to 7 geographical regions based on microsatellites yielded lower accuracy for the European and Middle Eastern populations. This can be again explained in terms of the proximity of these regions, both geographically and genetically (see Additional file 3: Table S2 and^22^). The Central/South Asian population is also genetically close to the European and Middle Eastern populations but both the MMD and STRUCTURE methods provided a reasonably accurate self-attribution for individuals from C/S Asia. The human 2,810 SNP genotypes data set predicts a similar pattern for the allele-frequency divergence between populations (Additional file 3: Table S3); Europe, Middle East and C/S Asia are the genetically closest populations. Self-attribution of C/S Asian individuals based on the MMD method is, however, poorer for the 2,810 SNP data set than for microsatellites (compare Additional file 2: Figs. S1 and S2). Self-attribution accuracy increased when using the 659,276 SNP dataset. In this case, self-attribution was very accurate ($>90 \%$) for all 7 regions with only a $10\%$ chance that individuals from C/S Asia and Middle East are wrongly attributed. This can again be ascribed to the relatively high genetic and geographical proximity between individuals from these regions (see Additional file 3: Table S4). Self-attribution of humans to 53 populations using the 659,276 SNP dataset was highly accurate for most populations (overall accuracy of $73\%$). Populations that were poorly self-attributed are again genetically and geographically close to those populations to which they were wrongly attributed.

The self-attribution accuracy achieved for *P. californicus* with the MMD method is, within statistical error, comparable to that obtained in Ref.^62^ (see Additional file 4). The self-attribution accuracy of breast cancer tumours is relatively low ($63\%$ overall correct self-attribution, see Additional file 5). The fact that wrongly attributed samples are evenly attributed to the two wrong subtypes is likely due to the similarity between subtypes (see Additional file 3: Table S5). Our hypothesis is that the self-attribution accuracy could significantly improve by extending the dataset with more samples to describe the subtypes.

The fact that the MMD method uses the Hamming distance between genotypes contrasts with many other assignment methods that rely on allele frequencies^3,5,14,15,19,37,38,50,51,70–73^. This includes a range of methods that use frequency-based genetic distances that differ from the Hamming distance^51,74^. Using the Hamming distance makes the MMD method intrinsically faster than frequency-based methods. Indeed, the runtime complexity of frequency-based methods increases linearly with the number of loci in the multilocus genotypes. In contrast, the computational complexity of the MMD method increases with the Hamming distance (see Methods). Since the Hamming distance is typically smaller than the number of loci (see some examples in Additional file 2: Fig. S13), this represents a significant speed improvement.

Frequency-based assignment methods (including those using genetic distances) traditionally quantify the similarity between the individuals and sources in terms of a scalar quantity (e.g. a genetic distance or the value of a likelihood function, see Methods). In contrast, the MMD describes the similarity between individuals and sources in terms of the probability distribution of the distance (more explicitly, it uses the cumulative distribution function $F_{u,s}(\lambda )$ of the Hamming distance, as described in the Methods). Measures of similarity used in traditional methods could be regarded as summary statistics of the distribution function. For instance, for unlinked loci, the likelihood function used by some frequency-based methods^3,37,51,70,74,75^ corresponds to the probability that the Hamming distance between an individual and a source is zero (see Additional file 6). In general, the distance probability distribution gives a more complete description of the similarity between individuals and sources than specific characteristics of the distribution. The *Campylobacter* dataset is an interesting example in which using the whole distribution is convenient since it is often bimodal and a description in terms of a single statistical measure might not be appropriate (see Additional file 2: Fig. S13).

The MMD method assumes that the genetic profile of populations is defined by the genotypes of the individuals sampled from each source. In this respect, it is similar to some distance and frequency-based methods that determine the allele frequencies straight from the observed genotypes^3,15,50,51,72–74^. The frequency-based methods show a certain arbitrariness when an allele is present in the individual to be assigned but it was not observed in any of the sources. In order to make sure that the individual is assigned to a source, some methods set the frequency of the missing allele in the sources to a small value^76^ or to a value given by the inverse of a beta distribution^25^. In the MMD method, a missing allele will simply contribute one unit to the Hamming distance between the individual and all sources. The MMD method implicitly assumes that those alleles that are missing in all sources do not bring any relevant information for source attribution.

Strictly speaking, the allele probabilities of a population cannot be fully determined from the observed allele frequencies in a sample (i.e. the sample will typically not cover the whole population and observed allele frequencies only give an approximate representation of the genetic profile of the population). To circumvent this problem, several frequency-based assignment methods use Bayesian approaches to model the allele probability distributions of the populations^5,14,19,37,38,51,70,71^. It has been reported that source attribution based on Bayesian methods often outperforms plain frequency-based methods^51^. Extending the MMD method by using Bayesian methods to infer genotypes within sources is a possibility that could be explored in the future. However, since we are now immersed in the big data era, to take advantage of this it is likely that a better strategy to ensure high assignment accuracy can be achieved exploiting non-Bayesian techniques such as the MMD method.

The largest differences between MMD and STRUCTURE self-attribution results were observed for sheep *Campylobacter* isolates (STRUCTURE does poorly, see Fig. 2(a)) and humans from C/S Asia based on 2,810 SNPs (MMD does poorly, see Fig. S2). We hypothesise that these differences could be associated with two factors. On the one hand, STRUCTURE uses sophisticated methods to infer the allele probabilities of sources. In principle, such probabilities could give a more precise characterisation of sources than those used in the MMD method which is just based on observed genotypes. On the other hand, even small errors in the estimate of the allele probabilities for STRUCTURE lead to an attribution error that increases faster with the number of loci than that of the MMD method (according to our arguments in Additional file 6, this is expected for any method using a likelihood function to measure the similarity between individuals and sources^3,5,14,15,19,37,38,48,51,70–74^). Based on these considerations, one would expect a lower accuracy for the MMD method when using genotypes with a relatively small number of loci (e.g. for our 2,810 human SNPs example). In contrast, for extended genotypes, the error of the likelihood function used by STRUCTURE can become large and this may result in a poor attribution accuracy compared to that of the MMD method.

ADMIXTURE also uses a likelihood function to estimate the ancestry and allele frequencies and this might explain its lower self-attribution accuracy compared to MMD for the human 659,276 SNP dataset. In spite of that, ADMIXTURE gave a rather accurate self-attribution for this dataset and deviations from a perfect self-attribution can be explained in terms of geographic and genetic proximity between regions (e.g. the probabilities for attribution of European individuals to C/S Asia and Middle East).

In general, the performance of any method might depend on specific details of data sets (e.g. distribution of populations within the genotype space and the level of intermixing). Identifying the specificities of data sets that would favour one source attribution method over another in terms of accuracy can be achieved on a case by case basis employing training datasets as we have done here for self-attribution. However, this might require laborious analysis of genotypes to find specific features.

A central assumption of assignment methods is that the set of sampled sources includes the true population of the individual to be assigned. Accordingly, individuals are assigned to at least one source even if there is a big difference between the individual and all sources. The MMD method is not different in this respect. In order to assess the likelihood that the true population of origin of an individual has been sampled, one should use an exclusion test^51^. We applied the threshold exclusion method proposed in Ref.^5^ for STRUCTURE to the MMD attribution for human genotypes with 659,276 SNPs and *Campylobacter* genotypes with 25,937 SNPs (see Additional file 7). The method only assigns an individual to a source if the attribution probability $p_{u,s}$ is above a threshold *T*. We found low exclusion rates for regions in the human dataset but exclusion was significant for the *Campylobacter* example even for self-attribution tests in which sources were definitely sampled. To understand the high exclusion rate for *Campylobacter* isolates, one should bear in mind that exclusion based on the threshold method does not necessarily imply that the source of the individual to be assigned has not been sampled. Instead, it might be a signature of a low genetic differentiation between sources. Consider, for instance, two genetically similar sources. The probability that an individual from one of the sources is attributed to any of the two sources will be around 1/2. Despite the fact that the source of the individual was definitely sampled, a threshold method will exclude both sources unless the threshold is very low (i.e. $T < 1/2$). When sources are not completely different to each other, it makes sense to assign individuals to several sources with certain probability rather than excluding sources with low assignment probability. For instance, the probabilistic assignment to several sources done by the MMD method should be the best way to capture the uncertainty in inference of source of infections (e.g. when investigating the source of campylobacteriosis). In contrast, assignment to a single source may be required in other applications such as parentage assignment^77^.

The optimal strategy for selecting loci for humans using either SNPs or microsatellites is S2 (targeting loci with high between-sources allele diversity) while for *Campylobacter* using cgSNPs is S1 (high within-source allele diversity). This difference is due to features within each of the datasets. Based on condition C1 given above that requires high allele diversity between sources, one would naively expect a more accurate attribution when loci with high between-source diversity are targeted (i.e. when using strategy S2). This is indeed the case for source attribution of humans. In contrast, strategy S1 performs marginally better for source attribution of *Campylobacter* isolates. In fact, loci with high within-source diversity in *Campylobacter* genotypes also have high between-source diversity (see Additional file 2: Fig. S6) and are less redundant than those with high between-source diversity (Additional file 2: Fig. S7). For this data set, a high diversity within sources combined with high diversity between sources seems to be a key factor for source attribution. This suggests that a high between-source diversity is necessary in general to distinguish different sources but it is not sufficient to ensure a high-quality source attribution. Based on this and given the formal similarity of the entropy between sources and informativeness^21^ explained in Methods, our results suggest that targeting loci with high informativeness (similar to S2) will not always be optimal compared to S1.

Strategy S3 (reordering loci targeted by strategies S1 and S2) did not bring a significant improvement on S1 or S2 for any of the examples considered here. This suggests that the redundancy of the loci targeted with strategies S1 and S2 does not play an important role in source attribution for these examples. We expect that the relative performance of S3 compared to S1 and S2 will depend on the data set. For instance, S3 could improve on S1 and S2 for data sets with high linkage disequilibrium. For cases in which linkage disequilibrium plays a crucial role, one could devise selection strategies with lower computational complexity than strategy S3. For instance, one could filter out one of the two loci in a pair when such a pair is in high linkage disequilibrium^77^. Strategies focusing on pairs of loci (e.g.^77^) should be computationally faster to apply than S3 but they are expected to be less accurate than S3 in datasets with high linkage disequilibrium.

For *Campylobacter* isolates, we have shown that it is sufficient to use the 10 cgSNPs with the highest within-source entropy to achieve a self-attribution accuracy of $\sim 70\%$ that is comparable to that obtained with 25,937 cgSNPs (Fig. 4(a)). In contrast, a much slower increase of the self-attribution accuracy was observed for the human data sets (based on the 659,276 SNPs dataset, one needs more than 1,000 SNPs for the attribution accuracy to saturate). The reason for the slow increase is unclear. It appears there is a lack of loci with high discriminatory power in the human data sets. In fact, loci with high between-source diversity are scarce compared to the *Campylobacter* dataset even in the 659,276 SNPs dataset (compare panels (a) and (b) in Additional file 2: Fig. S6). This difference between human and *Campylobacter* genotypes might be because human SNPs are inherently less diverse than *Campylobacter* SNPs. Another possibility is that 659,276 human SNPs represent a small fraction of the human genome (3.2 GBases) that is perhaps not representative enough in terms of loci diversity (compared to 25,937 cgSNPs which is a larger fraction of the *Campylobacter* genome consisting of 1.8 Mbases). In any case, using 659,276 SNPs is sufficient to achieve highly accurate attribution for humans with the MMD method.

The increase of the self-attribution accuracy with the number of selected loci is also slower for the *P. californicus* and breast cancer examples compared to the *Campylobacter* example (see Additional files 4 and 5). For *P. californicus*, this can be explained by the extremely low between-source diversity of SNPs (Additional file 2: Fig. S6(c)). Due to this, individual SNPs do not efficiently distinguish between the north and south regions in this case even if there is a relatively high within-source diversity (i.e. condition C1 for accurate source attribution is not well satisfied). An accurate distinction between individuals from the north and south regions can only be achieved by combining $\sim 100$ SNPs; the particular strategy used to select these SNPs does not seem to play a crucial role. Loci diversity is also limited in the breast-cancer proteotypes but the fraction of loci with high between-source diversity is promising. Including more samples in the dataset could potentially enhance the loci diversity in such a way that high attribution accuracy could be achieved by targeting few informative loci. Irrespective of this, it is interesting that even with a limited number of samples, the MMD method already achieves a relatively high self-attribution accuracy using $\sim {500}$ proteomic loci.

The genetic profile of individuals and sources can be represented with a wide range of genetic markers including microsatellites, gene-based markers or SNPs^78^. Data consisting of genotypes which contain large enough sets of highly polymorphic markers will typically offer high discriminatory power. Following this, one can achieve similar attribution accuracies using relatively short multilocus genotypes containing highly polymorphic markers (e.g. microsatellites) or extended genotypes containing less diverse markers (e.g. SNPs). With the current genomic technologies it is becoming increasingly feasible to obtain large sets of SNPs from genomes of many individuals. Combining extended SNP genotypes and fast methods for source attribution such as the MMD provides a significant opportunity for the future of source attribution approaches. Similar arguments apply to other OMIC datasets which are becoming increasingly available, as illustrated in our cancer example.

## Conclusions

The MMD method is very fast, easy to use, suitable for a range of types of loci (e.g. SNP, cgMLST, microsatellite, proteomics loci, etc.) and provides similar assignment accuracies to other methods. The best method for determining the minimum set of loci for optimal attribution varies between datasets. It is therefore prudent to employ a number of methods on each dataset to decide which set of loci are optimal. Some of the locus selection methods can be very computationally intensive (greedy strategies such as S3) and may not be practical to be used in conjunction with current attribution methodologies which are relatively slow. In contrast, the performance of different locus selection strategies can be tested relatively fast with the MMD method. The methods described in this paper are relevant for multiple applications in the life sciences and although they have only been applied to DNA- and proteomics-based methods here, could potentially also be used on other OMIC datasets (e.g. metabolomics) to characterise populations.

## Methods

### *Campylobacter* infectious disease example

Whole genome sequenced *Campylobacter* isolates comprising 500 clinical isolates from human patients and 673 isolates from five food and animal sources were obtained : cattle (150), chicken (150), pig (130), sheep (150) and wild bird (93) (Suppl data file S1). PanSeq^79^ was used to construct a non-redundant pan-genome from all of the 1,173 genomes, using a seed genome and identifying regions of $\ge 1{,}000$ base pairs (bp) not found in the seed, but present in any other genome at $87\%$ sequence identity cut-off. Loci present in all genomes underwent multiple sequence alignment and were concatenated. This aligned sequence was used to identify SNPs ($n = {25{,}937}$ in the core genome of all isolates, see more details in Additional file 1: Suppl data file S1).

### Human evolution example

Assignment of human individuals was illustrated for three data sets with individuals from 7 different geographic regions of the world. The first dataset comprised 5,795 human individuals from 7 different regions (Africa, America, Central/South Asia, East Asia, Europe, Middle East and Oceania). The genotype of each individual was described by 645 microsatellite markers^22^ (Additional file 1: Suppl data file S2). The second dataset comprised 1,107 Individuals from the same 7 regions of the microsatellite data set and their genotypes were described by 2,810 SNPs^61^ (Additional file 1: Suppl data file S3). The third dataset comprised 938 humans from the same geographic regions available from the Human Genome Diversity Panel (HGDP). The genotype of each individual was described by 659,276 SNPs^60^ (Additional file 1: Suppl data file S4).

### Attribution methodology

The aim of source attribution is to estimate the probability $p_{u,s}$ that an individual of unknown origin, *u*, originates from a source *s* from a set ${{{\mathscr {S}}}}$ of sources. For haploid genotypes, the unknown individual is characterised by a set of *L* loci, $\mathbf{u}= \{u_{l}\}_{l=1}^L$. Here, $u_l$ denotes the allele of the individual *u* at locus *l*. The set of possible values taken by the alleles is denoted as ${{{\mathscr {A}}}}$. The genetic information of a source *s* is represented by $I_s$ multilocus genotypes; the genotype of an individual *i* in the source *s* is characterised by a set of *L* loci, $\mathbf{a}_{i,s}=\{a_{i,s,l}\}_{l=1}^L$. Methods will be described for haploid genotypes but they can be readily extended to diploid genotypes or descriptions of individuals in terms of feature vectors of any kind. In the diploid case, genotypes are characterised by a sequence of *L* loci, each with two alleles: $\mathbf{a}_{i,s}=\{ (a_{i,s,l_1}, a_{i,s,l_2}) \}$. This information can be encoded as a feature vector consisting of 2*L* elements which can be readily used by the MMD method. Alternatively, one can encode the information into a vector of *L* elements by replacing pairs $(a_{i,s,l_1}, a_{i,s,l_2})$ by a single value, as described in Additional file 1: Suppl file S3 for the 65,533 SNP human genotypes. A method to extract a feature vector from proteomic data is described in Additional file 5.

The source attribution probabilities are summarised by the distribution probability $p_s$ that a randomly chosen individual from a set of $I_u$ isolates of unknown origin (e.g. $I_u=500$ *Campylobacter* isolates in Fig. 1) is attributed to the source *s* on average. We assume that $p_s$ has an inherent uncertainty associated with the fact that the set of $I_u$ assigned genotypes is a sample of a larger population of genotypes. In order to estimate the uncertainty of $p_s$, we estimate its probability distribution by bootstrapping^80,81^ based on the source probabilities $\{p_{u,s}\}_{u=1}^{I_u}$ for the $I_u$ assigned genotypes. For a given source, *s*, bootstrapping was implemented as follows: (i) draw a random sample with replacement of $I_u$ elements from the set $\{p_{u,s}\}_{u=1}^{I_u}$. (ii) Calculate the sample mean, ${\bar{p}}_s$, of the selected values of $p_{u,s}$. (iii) Repeat steps (i) and (ii) $n_{\text {b}}$ times ($n_{\text {b}}=10^4$ in our calculations). This results in $n_{\text {b}}$ values of ${\bar{p}}_s$ that define our estimate for the distribution of $p_s$. The error bars in Figs. 1, 2, S1 and S2 correspond to $2.5\text {th}$ and $97.5\text {th}$ percentiles of the $p_s$ distribution.

A Monte-Carlo cross-validation strategy^65,66^ was used for self-attribution. More explicitly, $I_u$ individuals were randomly removed from each source population (testing or validation set) and they were attributed to the sources described by the remaining genotypes (learning or training set). The origing of the removed $I_u$ individuals is assumed to be unknown and the probability $p_s$ that any of them is attributed to source *s* (see Figs. 2, S1 and S2) is calculated by bootstrapping, as explained above for source attribution. The self-attribution accuracy is summarised in Fig. 4a, b in terms of the total self-attribution probability $p^{\text {sa}}$ defined as the mean over sources of the probability $p_s$ that individuals from each source are attributed to their source. The confidence interval of $p^{\text {sa}}$ is estimated by the mean over sources of the $2.5\text {th}$ and $97.5\text {th}$ percentiles of the correct self-attribution probability $p_s$ for each source.

For the *Campylobacter* and humans examples, $50\%$ of the samples were removed from the source to be tested for self-attribution (i.e. $I_u=I_s/2$). Details on the self-attribution analysis for *P. californicus* and breast cancer samples are given in Additional files 4 and 5, respectively.

#### The MMD method

The MMD method uses the multilocus genotypes $\mathbf{u}$ and $\mathbf{a}_{i,s}$ to determine the probability $p_{u,s}$ as follows:

(i) Calculate the Hamming distance^64^, $d_{\text {H}}(\mathbf{u},{\mathbf{a}}_{i,s})$, between the genotype of unknown origin and genotypes *i* in source *s*.
(ii) Obtain a score $\sigma _{u,s}$ which quantifies the proximity of $\mathbf{u}$ to source *s*. The calculation of $\sigma _{u,s}$ is based on the cumulative distribution function $F_{u,s}(\lambda )$ that gives the probability that the Hamming distance between $\mathbf{u}$ and any genotype of source *s* is smaller than $\lambda$ (see Additional file 2: Fig. S8). The proximity between $\mathbf{u}$ and each of the sources *s* is measured by the *q*-quantile $\lambda _{u,s}(q)$ corresponding to the distribution $F_{u,s}(\lambda )$. For a given probability *q*, the closest source to $\mathbf{u}$ is the one with the smallest value of $\lambda _{u,s}(q)$:
  1 $$\begin{aligned} \lambda _{\min }=\min _s \{\lambda _{u,s}(q)\}. \end{aligned}$$
  Once $\lambda _{\min }$ has been obtained, the score is calculated as $\sigma _{u,s}=F_{u,s}(\lambda _{\min })$, i.e. it is the probability that the Hamming distance of $\mathbf{u}$ to any source *s* is $\lambda _{\min }$ or smaller. This ensures that sources with high probability to be close to $\mathbf{u}$ are given a high score (see a graphical representation of the procedure in Additional file 2: Fig. S8).
(iii) Estimate the probability that $\mathbf{u}$ is attributed to source *s* as $p_{u,s}=\sigma _{u,s}/\sum _{s'\in {{\mathscr {S}}}}\sigma _{u,s'}$. Note that an individual $\mathbf{u}$ is necessarily attributed to at least one source by the methodology.

The Hamming distance can be calculated in times proportional to the Hamming distance itself^82^. Accordingly, the time complexity for attribution of an individual with MMD is $O(d_{\max } I_{\text {Tot}})$, where $d_{\max }$ is the maximum Hamming distance between the genotype of unknown origin and the genotypes used to describe sources and $I_{\text {Tot}} = \sum _{s\in {{{\mathscr {S}}}}} I_s$ is the total number of genotypes used to describe sources.

The probability *q* is a parameter of the model. In self-attribution tests, the optimal value of this probability was obtained for each source *s* as the value $q_*$ that maximises the probability $p_s$ that individuals are correctly attributed to their source (in some cases, $q_*$ can be defined as an interval of *q* where the maximum self-attribution probability is observed). Results of the correct self-attribution probability as a function of *q* are shown in Additional file 2: Figs. S9–S11. The optimal value/interval $q_*$ depends on the particular set of individuals set as unknown for self-attribution but it is relatively small in all the examples studied here (in most cases, $q_*<0.1$). This makes sense since one would expect that large differences in $\sigma _{u,s}$ for different sources would be mainly dictated by few genotypes that are closer to the individual $\mathbf{u}$ in its source. In particular, setting $q=0$ defines an extreme version of our algorithm with $\lambda _{\text {min}}=\min _{i,s} \{d_{\text {H}}(\mathbf{u},\mathbf{a}_{i,s})\}$. In this case, the score $\sigma _{u,s}$ is the proportion of genotypes in source *s* that are a distance $d_{\text {min}}$ from the individual to be assigned, $\mathbf{u}$. We checked that self-attribution accuracy is already high when we set $q=0$ in our examples. In general, however, $\sigma _{u,s}$ obeys the extremal value statistics of the Hamming distance for $q=0$ and might not be reliable enough if the number of genotypes, $I_s$, used to describe each source, *s*, is not large enough. When $I_s$ is not large enough and extended genotypes are used, individuals of unknown origin tend to be attributed to a single source *s* with probability $p_{u,s}=1$ (i.e. the condition $d_{\text {H}}(\mathbf{u},\mathbf{a}_{i,s})=d_{\text {min}}$ is only satisfied for one genotype).

For source attribution, *q* cannot be obtained through optimisation since the actual origin of individuals to be attributed is genuinely unknown. In this respect, it can be useful to do self-attribution with genotypes from source populations to estimate a suitable value of *q*. For instance, the source attribution results for human *Campylobacter* isolates shown in Fig. 1(b) correspond to $q=0.05$ which is the mean of the optimal self-attribution values, $q_*$, weighted by the number of isolates in each source (see Additional file 2: Fig. S9). In fact, source attribution is not very sensitive to the specific value of *q*, provided it is within the range in which self-attribution probability is high. Compare, for instance, the results for $q=0$ illustrated in Additional file 2: Fig. S12 with those for $q=0.05$ in Fig. 1b.

#### The STRUCTURE method

STRUCTURE is a Bayesian clustering model proposed to infer population structure and assign individuals to populations. Following previous works^8,11–13,83^, STRUCTURE was used to estimate $p_{u,s}$ by setting the number of clusters to be equal to the number of sources (e.g. $K=5$ for the *Campylobacter* example or $K=7$ for the humans attribution example). The population structure of the sources was assumed to be known (i.e. we set USEPOPINFO=1 and POPFLAG=1 for the source isolates). In contrast, the population structure of the $I_u$ isolates to be attributed was set as unknown with POPFLAG=0. The results presented are based on runs of $10^4$ MCMC steps following a burn-in period of $10^4$ iterations. The statistics of $p_s$ were obtained from $p_{u,s}$ as explained above for the MMD method.

#### The ADMIXTURE method

ADMIXTURE uses multilocus genotype data for efficient estimation of ancestry of unrelated individuals^40^. ADMIXTURE infers the ancestry of individuals in terms of the admixture proportion $h_{u,s}$ of the genome of individual $\mathbf{u}$ that originated from population *s*. In the supervised version of ADMIXTURE^48^, the ancestry of reference populations is determined by the genotypes of the individuals in the training set (i.e. all individuals except the $I_u$ individuals selected for the validation set). The admixture proportion $h_{u,s}$ for individuals in the validation set quantifies the proportion of their genotype originating from the reference population *s*. This can be regarded as a measure of genetic proximity between the individual $\mathbf{u}$ and population *s*, formally similar to the attribution probability $p_{u,s}$ estimated by the MMD method. Following this, our application of ADMIXTURE for source attribution uses a supervised analysis and assumes that the attribution probability is $p_{u,s}=h_{u,s}$.

### Information theory: loci diversity and redundancy

We quantify the allele diversity in terms of the Shannon entropy, a measure of the information (in bits) necessary to describe the uncertainty of random variables^57^. The Shannon entropy is increasingly used as a diversity index in ecology^84,85^ and population genetics^86–88^. In our application, the random variables are the alleles found in the genotypes of sources at a locus *l*. More explicitly, we consider the probability $\pi _{a,l,s}$ that an allele takes the value *a* at the locus *l* and $\pi _{a,l}^{\text {T}}=\sum _{s\in {{{\mathscr {S}}}}} q_s \pi _{a,l,s}$ which gives the allele probability pooled over sources. Here, $q_s=I_s/\sum _{s\in {{{\mathscr {S}}}}} I_s$ is the proportional weight of each source. The total allele diversity in a locus *l* is quantified by the Shannon entropy of the distribution $\pi _{a,l}^{\text {T}}$,

2 $$\begin{aligned} H_{l}^{\text {T}}=-\sum _{a \in {{{\mathscr {A}}}}} \pi _{a,l}^{\text {T}} {\mathrm {log}}_2 \pi _{a,l}^{\text {T}}. \end{aligned}$$

For example, the fact that the maximum number of alleles in a SNP is 4 (A, T, C and G) implies that the entropy $H_l^{\text {T}}$ could take any value between 0 (the same allele in all genotypes) and 2 bits (maximal diversity when each allele appears in 1/4 of the genotypes). As expected for any measure of allele diversity, the larger the number of alleles in a locus, the larger the Shannon entropy. Microsatellites^22^ or gene-based markers^24^ are characterised by larger sets ${{{\mathscr {A}}}}$ of possible alleles and can be more diverse than SNPs, i.e. they have larger values of $H_l^{\text {T}}$ which are mostly associated with a larger contribution of the diversity within-sources, $H_l^{\text {W}}$ (see Additional file 2: Fig. S6).

The entropy $H_l^{\text {T}}$ gives the allele diversity for subtypes in *all* the sources. The condition C1 given above for selection of informative markers, however, suggests that it is the allele diversity between sources the one that could play a major role on the assignment power of loci. Accordingly, we split the total entropy $H_l^{\text {T}}$ in two contributions^84,85^: One accounting for the diversity *within* sources,

3 $$\begin{aligned} H_{l}^{\text {W}}=-\sum _{a \in {{{\mathscr {A}}}}} \sum _{s\in {{{\mathscr {S}}}}} q_s \pi _{a,l,s} \text {log}_2 \pi _{a,l,s}, \end{aligned}$$

and another measuring the diversity *between* sources,

4 $$\begin{aligned} H_{l}^{\text {B}}=H_{l}^{\text {T}}-H_{l}^{\text {W}}. \end{aligned}$$

Basic algebraic manipulations show that $H_{l}^{\text {B}}$ is formally similar to the informativeness introduced in^21^. Our interpretation of $H_{l}^{\text {B}}$ is, however, slightly different to that proposed in^21^ since we derived it as an index to distinguish sources rather than as a measure of the information gained when adding new loci to the selection used for attribution.

#### Mutual information and redundancy of loci

The source attribution discriminatory power of a set of *n* loci is typically not *n* times larger than the discriminatory power of each isolated locus. This is due to the fact that loci are not statistically independent, i.e. there is some redundant information when considering several loci. The concept of loci redundancy was used in this work with two aims: To select pairs of loci with low redundancy in strategy S3 and to assess the extent to which strategies S1 and S2 satisfy the condition C2 of low redundancy.

The elementary quantity in our estimates of loci redundancy is the mutual information between pairs of loci. Given a pair of loci, $(l,l')$, it is defined as^57,87,88^:

5 $$\begin{aligned} I_{l,l'}=\sum _{a,a'\in {{{\mathscr {A}}}}} \pi _{a,l,a',l'} \text {log}_2 \frac{\pi _{a,l,a',l'}}{\pi _{a,l} \pi _{a',l'}}. \end{aligned}$$

Here, $\pi _{a,l,a',l'}$ is the joint probability distribution for alleles in locus *l* and $l'$. Within the context of population genetics, $I_{l,l'}$ has been used to quantify the linkage disequilibrium between loci *l* and $l'$^87^. The mutual information takes values $0\le I_{l,l'} \le \min \{H_l,H_{l'}\}$. In particular, it is null, i.e. $I_{l,l'}=0$, when the allele distributions of the two loci are independent. In general, $I_{l,l'} \le I_{l,l}$ meaning that a locus contains as much information about itself as any other locus can provide. In other words, a locus *l* is maximally redundant with respect to itself. In the case $l=l'$, the mutual information coincides with the Shannon entropy, $H_l=I_{l,l}$. Within the context of this work, mutual information is used as a measure of the linkage disequilibrium between the pairs of loci *l* and $l'$. In fact, $I_{l,l'}$ is proportional to the widely-used^56^ measure for linkage disequilibrium, $r^2$, in the limit of $\pi _{a,l,a',l'} \ll 2 \pi _{a,l} \pi _{a',l'}$^87,89^. Despite the similarity with classical measures such as $r^2$, the mutual information gives an intuitive interpretation of linkage disequilibrium and, as discussed in^87,89,90^, has some other advantages over classical measures.

For our particular application, $I_{l,l'}$ allows us to naturally define a measure of loci redundancy relevant to strategy S3. The redundancy $R_n$ of the *n*th locus added to a list of $n-1$ previously selected loci is given by the following formula:

6 $$\begin{aligned} R_n=\max _{l=1,2,\ldots ,n-1} \{s_{n|l}\}. \end{aligned}$$

Here, $s_{n|l}=\frac{I_{n,l}}{H_{n}}$ is the reduction in uncertainty of a locus $l=1,2,\dots ,n-1$ when the *n*th locus is added to the set used for source attribution. The definition of $s_{n|l}$ and the whole redundancy analysis is restricted to loci with $H_n>0$; loci with $H_n=0$ consist of a single allele and are excluded from the analysis since they have a null discriminatory power. From Eq. (6), $R_n$ can be interpreted as the maximal reduction in uncertainty achieved when adding the *n*th locus to the list of selected loci. By definition, $0 \le R_n \le 1$. The case $R_n=0$ corresponds to the smallest possible redundancy of locus *n* and is observed when the allele distribution at such locus is statistically independent of the allele distribution at any of the previously selected loci *l*. The case $R_n=1$ indicates that $s_{n|l}=1$ for at least one of the previously selected loci, thus indicating that the allele distribution of the newly added locus, *n*, is identical to the allele distribution of at least one of those that were previously selected. In this case, the locus *n* would not contribute to enhance the discriminatory power of the set of selected loci.

## Supplementary information

Supplementary Information 1.

Supplementary Information 2.

Supplementary Information 3.

Supplementary Information 4.

Supplementary Information 5.

Supplementary Information 6.

Supplementary Information 7.

## Data Availability

Data used in this work are available from https://figshare.com/s/726d493387b501c4b70a. An executable version of the MMD program can be downloaded from https://figshare.com/s/125861c0a0499ff3101b. The software is based on the MMD R package developed in this project.
