# Supplementary material for: Mining whole genome sequence data to efficiently attribute individuals to source populations
Source: Sci Rep. 2020 Jul 22;10:12124. doi: 10.1038/s41598-020-68740-6 (PMC7376179; doi:10.1038/s41598-020-68740-6)
Supplement: Supplementary file 1 — Supplementary Information 1. [file 41598_2020_68740_MOESM1_ESM.pdf]

Mining Whole Genome Sequence data to efficiently attribute individuals to source populations

Additional file 1: Supplementary data files

Francisco J. Pérez-Reche, Ovidiu Rotariu, Bruno S. Lopes, Ken J. Forbes and Norval J.C. Strachan

The data sets for the six examples studied in this work are available as compressed ZIP files from

https://figshare.com/s/726d493387b501c4b70a

For each example, the corresponding ZIP file contains:

- Two text files that are used by the MMD program: A file with extension *.pop which lists the population corresponding to each genotype and a file with the same name but in comma-separated values (csv) format which contains the genotypes (or proteotype for the breast cancer example). *Loci must be in integer format* for our implementation of the MMD method in R.
- A directory called Preparing_data_for_MMD with a script to prepare the data with the format needed by the MMD program.

Supplementary data file S1, Campylobacter_25937SNP.zip - *Campylobacter* data

File with information about isolates

The file Campylobacter_Isolates_Accession.xlsx lists the 1173 *Campylobacter* isolates used in this example. The source, genome accession number, isolation date and sequence type (ST) are given for each isolate. Accession numbers are missing for two isolates from wild bird (they were originally downloaded from PubMLST but are not currently available). Genomes for these isolates are available from the authors by request.

Files for the MMD program

The files used by the MMD program are Campylobacter_25937SNP.csv and Campylobacter_25937SNP.pop. The Campylobacter_25937SNP.csv file contains genotypes consisting of 25 937 cgSNPs for the 1173 *Campylobacter* isolates. Each row in the file gives the genotype of one isolate with the following format:

SNP_1, SNP_2, ….., SNP_{25937}

SNPs can take values 1, 2, 3, 4 which correspond to the nucleotides A, T, G and C. Missing loci in the list of SNPs are coded by a negative number that is different for each row (i.e. different for each genotype). In this way, missing loci contribute to an increase of the Hamming distance between pairs of genotypes even if they are missing from both genotypes.

The file Campylobacter_25937SNP.pop consists of 1173 lines specifying the host name for each *Campylobacter* isolate. The host names are Human, Cattle, Chicken, Pig, Sheep and WB.

Files to prepare data for the MMD program
The directory Preparing_data_for_MMD contains the script Data_Campylobacter_25937SNP.R (R software (*1*)) which uses the files phylip_name_conversion.txt and snp.phylip generated by PanSeq (*2*) to build the MMD files Campylobacter_25937SNP.csv and Campylobacter_25937SNP.pop.

Supplementary data file S2, Human_645microsatellite.zip - Human microsatellite data

This dataset is an adaptation of the data used in Ref. (*3*) to the format needed for the MMD software.

Files for the MMD program
The ZIP file contains the files Human_645microsatellite.csv and Human_645microsatellite.pop. The file Human_645microsatellite.csv contains diploid genotypes with 1290 loci (2x645 microsatellites) for 5418 human individuals. Each row gives the genotype of one individual with the following format:

$$\text{Microsatellite}_1, \text{Microsatellite}_2, \ldots., \text{Microsatellite}_{1290}$$

Different integer numbers codes different microsatellites. As in Suppl. data file S1, missing alleles are indicated by a different negative number for each row.

The file Human_645microsatellite.pop lists the region of each individual. The names of the regions are AFRICA, AMERICA, CENTRAL_SOUTH_ASIA, EAST_ASIA, EUROPE, MIDDLE_EAST and OCEANIA.

Files to prepare data for the MMD program
The MMD files were obtained from the pembertonEtAl2013.MS5795.stru file from Ref. (*3*) (File S1 in the Supporting Information at https://www.g3journal.org/content/3/5/891.supplemental). The original data file pembertonEtAl2013.MS5795.stru is formatted for STRUCTURE (*4*) and was converted to the MMD format with the Mathematica (*5*) notebook Preparing_Data_Human_645microsatellite.nb available in the directory Preparing_data_for_MMD. The original data contains Latino and Afro European individuals which were neglected in our analysis for consistency with other examples on human populations (Human_2810SNP.zip and Human_659276SNP.zip, see below) which do not contain data on Latino and Afro European populations.

Supplementary data file S3, Human_2810SNP.zip - Human 2810 SNP data

This dataset is an adaptation of the data used in Ref. (*6*) to the format needed for the MMD software.

Files for the MMD program

The ZIP file contains two files: Human_2810SNP.csv and Human_2810SNP.pop. The Human_2810SNP.csv file contains diploid genotypes with 5620 loci for 1107 human individuals. Each row gives the genotype of one individual with the following format:

$$locus_1, locus_2, …., locus_{5620}$$

Loci can take values 1, 2, 3 and 4 which correspond to the nucleotides A, T, G and C, respectively. Each locus gives the allele of one of the copies in a pair of alleles (therefore, there are twice as many loci as SNPs). Missing loci are coded by a negative number that is different for each row (i.e. different for each genotype).

The file Human_2810SNP.pop lists the region of each individual. The names of the regions are AFRICA, AMERICA, CENTRAL_SOUTH_ASIA, EAST_ASIA, EUROPE, MIDDLE_EAST and OCEANIA.

Files to prepare data for MMD the program

The directory Preparing_data_for_MMD contains the Mathematica (*5*) notebook Preparing_Data_Human_2810SNP.nb used to build the Human_2810SNP.csv and Human_2810SNP.pop MMD files. The input for the notebook is the file phased_HGDP+India+Africa_ 2810SNPs-regions1to36.stru downloaded from https://rosenberglab.stanford.edu/diversity.html (dataset referred as "SNP data HGDP+India+Africa 2011 SNP data" in the link).

Supplementary data file S4, Human_659276SNP.zip - Human 659 276 SNP data

This dataset is an adaptation of the data used in Ref. (*7*) to the format needed for the MMD software.

Files for the MMD program

The ZIP file contains three files: Human_659276SNP.csv and Human_659276SNP.pop and Population_names_938.pop. The Human_659276SNP.csv file contains genotypes with 659276 diploid SNPs for 938 human individuals. Each row gives the genotype of one individual with the following format:

$$locus_1, locus_2, …., locus_{659276}$$

Loci take values {1,2,…,10} which represent pairs of alleles. The conversion is given by the following table:

AA	AT, TA	AG, GA	AC, CA	TT	TG, GT	TC, CT	GG	GC, CG	CC
1	8	5	9	3	7	6	2	10	4

Missing loci are coded by a negative integer that is different for each individual. Replacing pairs of alleles by a single number as indicated in the table leads to genotypes of length equal to the number of SNPs. We checked that source attribution based on the coding of pairs of alleles is as accurate as that achieved by coding each individual copy A, T, G, C by 1, 2, 3, 4 (the later coding leads to genotypes of 1 318 552 loci which can also be handled by the MMD program).

The file Human_659276SNP.pop lists the region of each individual. The names of the regions are AFRICA, AMERICA, CENTRAL_SOUTH_ASIA, EAST_ASIA, EUROPE, MIDDLE_EAST and OCEANIA. The file Population_names_938.pop lists the population of origin of each individual. There are 53 populations in total.

Files to prepare data for the MMD program

The directory Preparing_data_for_MMD contains the R script (*1*) Prepare_Data_Human_659276SNP.R used to build the files Human_659276SNP.csv and Human_659276SNP.pop and Population_names_938.pop. The R script uses the following files:

- HGDP_FinalReport_Forward.txt: Contains diploid genotypes for the 1043 samples used in our analysis. The file can be accessed from http://www.hagsc.org/hgdp/files.html.
- HGDP1066.txt and HGDP938.txt which link individuals used in the analysis to their population and region. These files are included in the Preparing_data_for_MMD directory.

Supplementary data file S5, Pcalifornicus_3699SNP.zip - Giant Californian sea cucumber *Parastichopus californicus* data

This dataset is an adaptation of the data used in Ref. (*8*) to the format needed for the MMD software.

Files for the MMD program

The ZIP file contains three files: Pcalifornicus_3699SNP.csv and Pcalifornicus_3699SNP.pop. The Pcalifornicus_3699SNP.csv file contains genotypes with 7398 loci for 717 human individuals. Each row gives the genotype of one individual with the following format:

$$locus_1, locus_2, …., locus_{7398}$$

Loci can take values 1, 2, 3, 4 which correspond to the nucleotides A, T, G and C. Each locus gives the allele of one of the copies in a pair of alleles (therefore, there are twice as many loci as SNPs). Missing loci are coded by a negative number that is different for each row (i.e. different for each genotype). The file Pcalifornicus_3699SNP.pop lists the region (North or South) of each individual.

Files to prepare data for the MMD program

The directory Preparing_data_for_MMD contains the R script (*1*) Prepare_Data_Pcalifornicus_3699SNP.R used to build the files Pcalifornicus_3699SNP.csv and Pcalifornicus_3699SNP.pop. The R script converts to the MMD format the SNP genotypes file filtered_3699snps_californicus.vcf available from (*8*) at https://datadryad.org/stash/dataset/doi:10.5061/dryad.db6177b.

Supplementary data file S6, Breast_Cancer_proteome.zip – Breast cancer proteomic data

This dataset is an adaptation of the proteomic data used in Ref. (*9*) to a format suitable for the MMD software.

Files for the MMD program

The ZIP file contains two files: Breast_Cancer_proteome.csv and Breast_Cancer_proteome.pop. The Breast_Cancer_proteome.csv file contains genotypes with 65533 loci for 40 human individuals. Each row gives the genotype of each of the samples with the following format:

$$locus_1, locus_2, ..., locus_{65533}$$

Loci can take values 0 or 1, corresponding to zero or positive values for the mass spectrum intensity, respectively. The Breast_Cancer_proteome.pop file list the cancer subtype of each sample. Subtypes are ERPR, Her2 and TN.

Files to prepare data for the MMD program

The directory Preparing_data_for_MMD contains the R script (*1*) Preparing_Data_Breast_Cancer_Proteome.R used to build the files Breast_Cancer_proteome.csv and Breast_Cancer_proteome.pop. The script uses the mass spectrum intensity from the file Datapeptides_Supp1.csv which was adapted to CVS format from the file Datapeptides_Supp1.xls available from Ref. (*9*) at https://www.nature.com/articles/ncomms10259#s1.

1. R Core Team, *R: A Language and Environment for Statistical Computing* (R Foundation for Statistical Computing, Vienna, Austria, 2015; https://www.r-project.org/).

2. C. Laing, C. Buchanan, E. N. Taboada, Y. Zhang, A. Kropinski, A. Villegas, J. E. Thomas, V. P. Gannon, Pan-genome sequence analysis using Panseq: an online tool for the rapid analysis of core and accessory genomic regions. *BMC Bioinformatics*. **11**, 461 (2010).

3. T. J. Pemberton, M. DeGiorgio, N. A. Rosenberg, Population Structure in a Comprehensive Genomic Data Set on Human Microsatellite Variation. *G3 GenesGenomesGenetics*. **3**, 891–907 (2013).

4. J. K. Pritchard, M. M. Stephens, P. Donnelly, Inference of population structure using multilocus genotype data. *Genetics*. **155**, 945–959 (2000).

5. Wolfram Research, Inc, *Mathematica, Version 12.1* (Champaign, Illinois, 2020; https://www.wolfram.com/mathematica).

6. L. Huang, M. Jakobsson, T. J. Pemberton, M. Ibrahim, T. Nyambo, S. Omar, J. K. Pritchard, S. A. Tishkoff, N. A. Rosenberg, Haplotype variation and genotype imputation in African populations. *Genet. Epidemiol.* **35**, 766–780 (2011).

7. J. Z. Li, D. M. Absher, H. Tang, A. M. Southwick, A. M. Casto, S. Ramachandran, H. M. Cann, G. S. Barsh, M. Feldman, L. L. Cavalli-sforza, R. M. Myers, Worlwide Human Relationships Inferred from Genome-Wide Patterns of Variation. *Science*. **319**, 1100–1104 (2008).

8. A. Xuereb, L. Benestan, É. Normandeau, R. M. Daigle, J. M. R. Curtis, L. Bernatchez, M.-J. Fortin, Asymmetric oceanographic processes mediate connectivity and population genetic structure, as revealed by RADseq, in a highly dispersive marine invertebrate (*Parastichopus californicus*). *Mol. Ecol.* **27**, 2347–2364 (2018).

9. S. Tyanova, R. Albrechtsen, P. Kronqvist, J. Cox, M. Mann, T. Geiger, Proteomic maps of breast cancer subtypes. *Nat. Commun.* **7**, 1–11 (2016).
