# Supplementary material for: Mining whole genome sequence data to efficiently attribute individuals to source populations
Source: Sci Rep. 2020 Jul 22;10:12124. doi: 10.1038/s41598-020-68740-6 (PMC7376179; doi:10.1038/s41598-020-68740-6)
Supplement: Supplementary file 2 — Supplementary Information 2. [file 41598_2020_68740_MOESM2_ESM.pdf]

Mining Whole Genome Sequence data to efficiently attribute individuals to source populations

Additional file 2: Supplementary figures

Francisco J. Pérez-Reche, Ovidiu Rotariu, Bruno S. Lopes, Ken J. Forbes and Norval J.C. Strachan

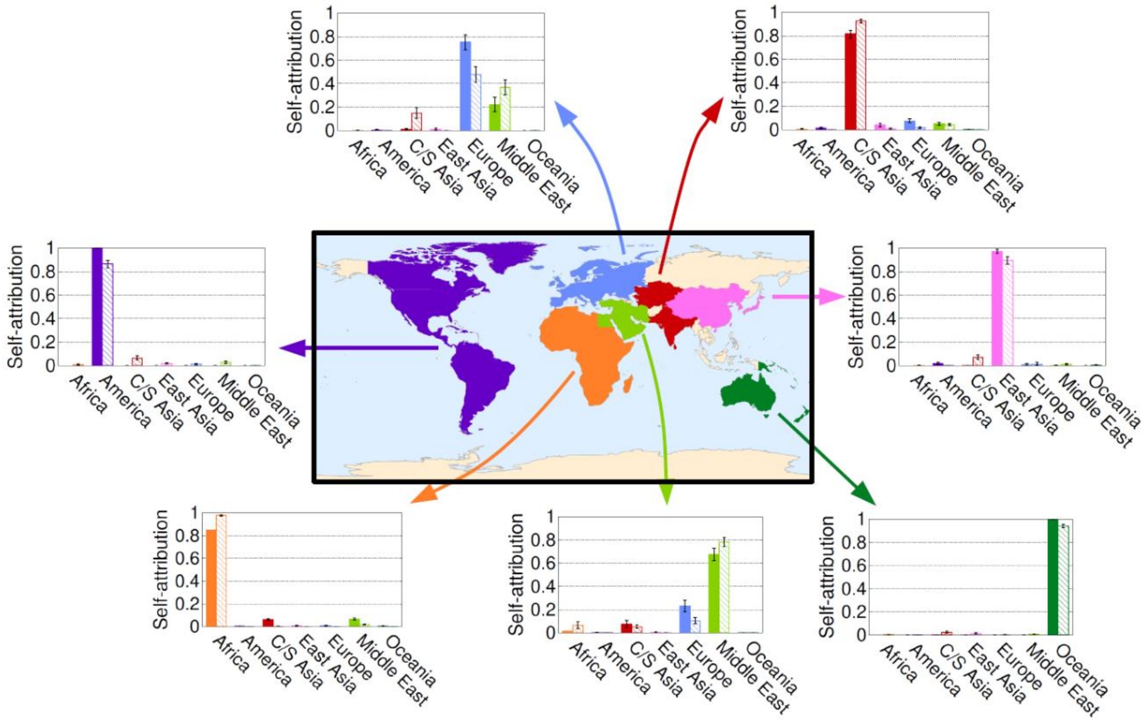

Fig S1. Self-attribution of humans characterised by 645 microsatellite genotypes. Bar charts show the probability distribution p_s. For a given source (region) s, p_s gives the probability that any individual from the region indicated in the map is attributed to s. Solid and hatched bars show the results obtained with the MMD method and STRUCTURE, respectively.

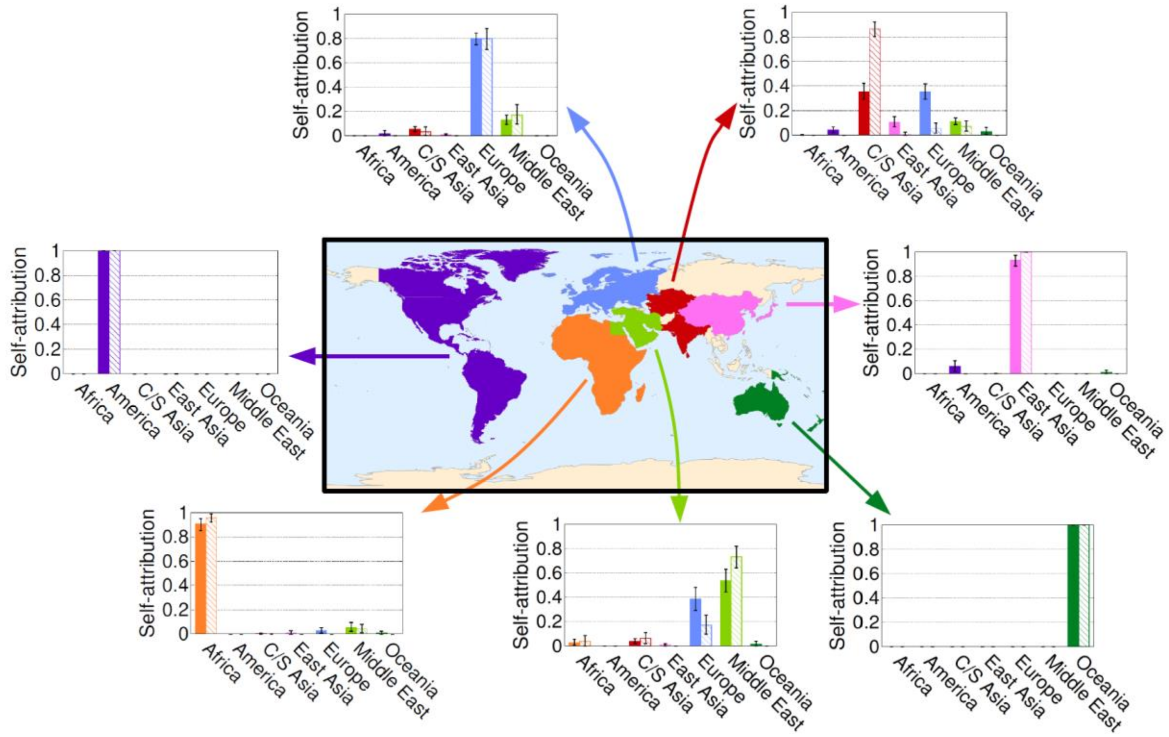

Fig S2. Self-attribution of humans characterised by 2 810 SNP genotypes. Bar charts show the probability distribution p_s. For a given source (region) s, p_s gives the probability that any individual from the region indicated in the map is attributed to s. Solid and hatched bars show the results obtained with the MMD method and STRUCTURE, respectively.

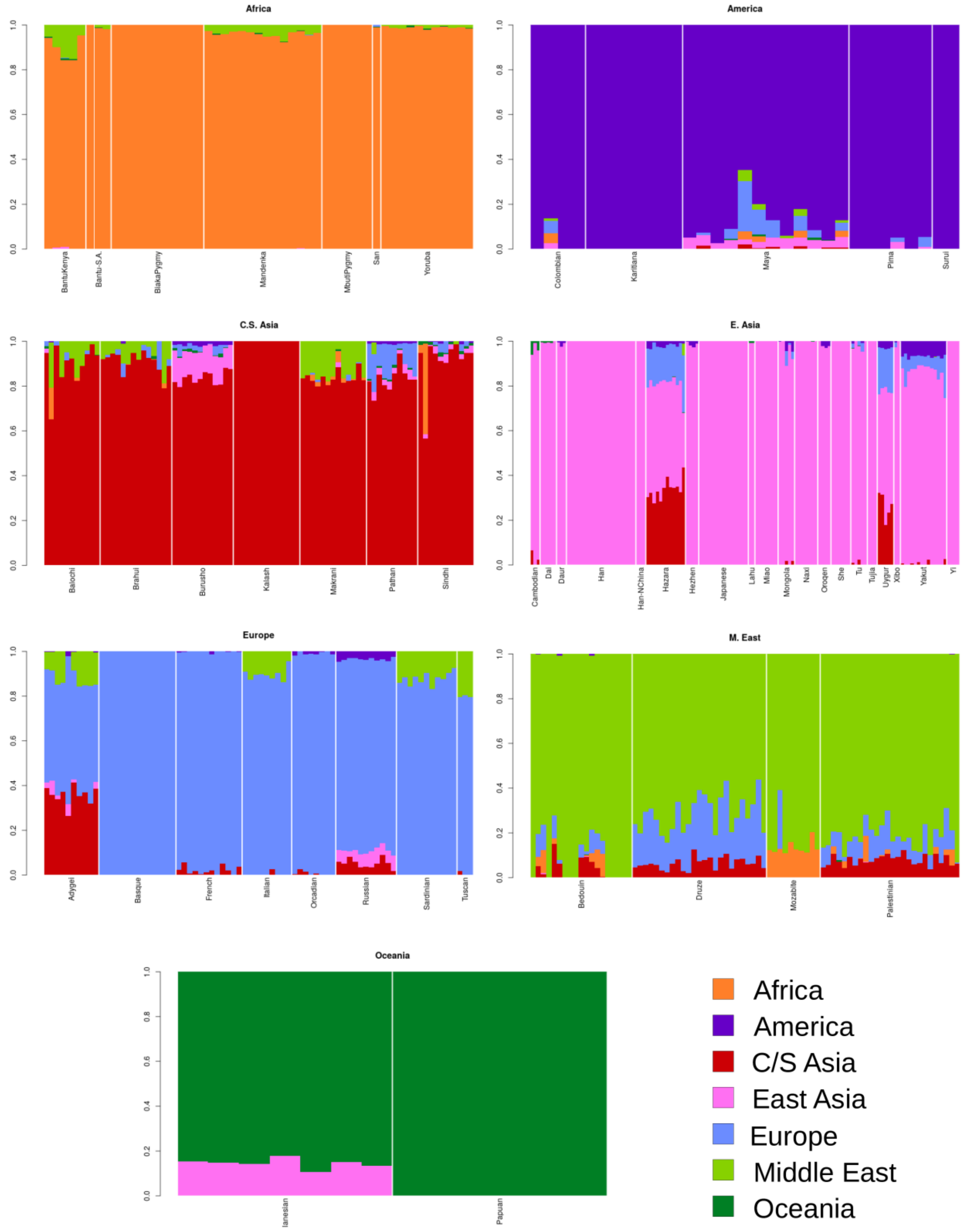

Fig S3. Supervised ADMIXTURE analysis of ancestry based on 659 271 SNP genotypes. Each panel shows the inferred admixture of 50% of individuals u selected from the geographical region indicated in the title of the panel. Each individual u is indicated by a vertical line, which is partitioned in segments of different colours that represent the admixture proportion $h_{u,s}$ of the individual from region s. The correspondence between regions and colours is given by the legend. Vertical white lines separate individuals from different populations.

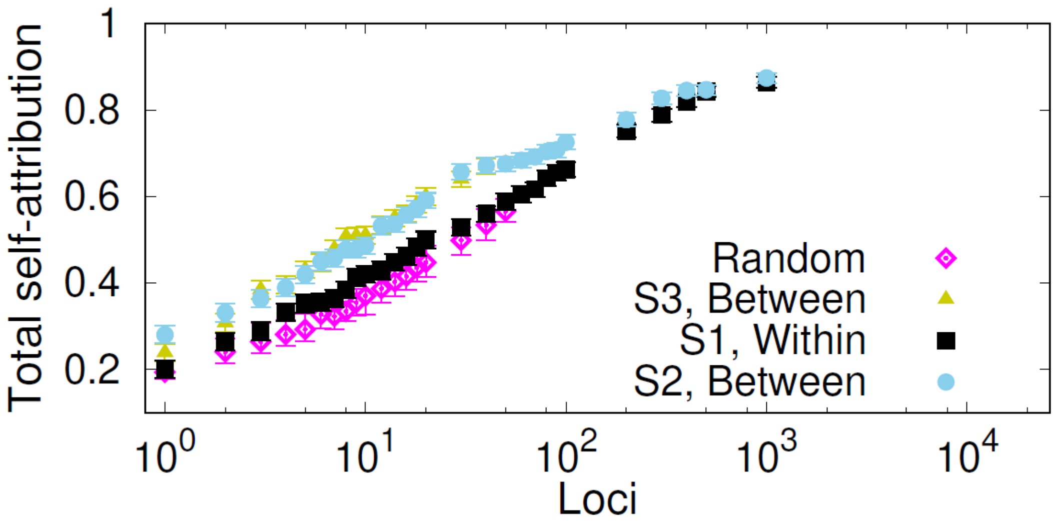

Fig S4. Selection of markers for self-attribution of humans based on 645 microsatellite genotypes. Symbols show the self-attribution probability p^{sa} that individuals from any of the 7 regions in the data set are correctly attributed to their region. The probability is plotted as a function of the number of SNPs selected at random and with strategies S1 (loci ranked in decreasing within-source diversity), S2 (loci ranked in decreasing between-source diversity) and S3 (reordering the loci ranking of S2 to reduce loci redundancy).

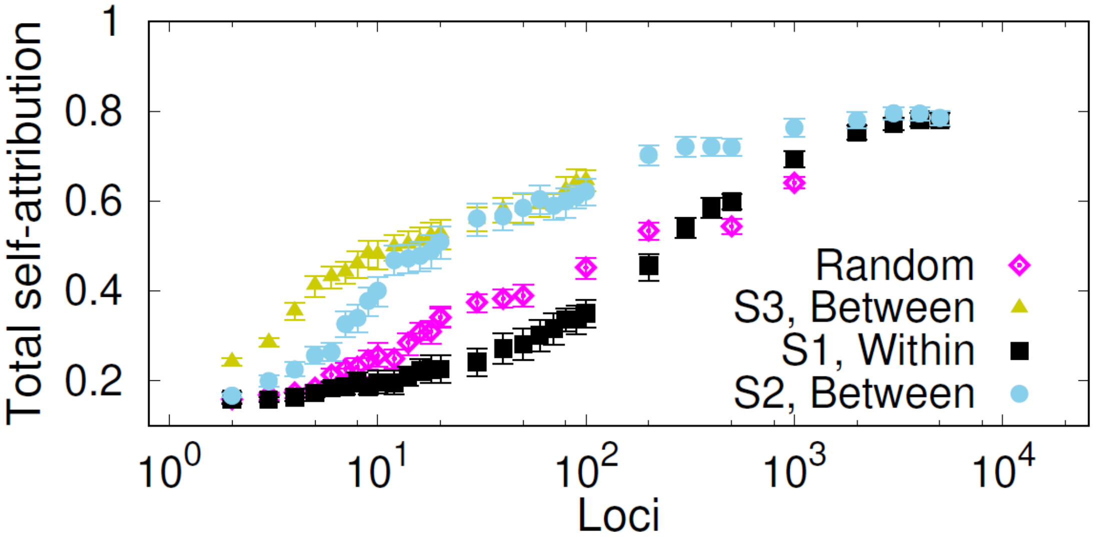

Fig S5. Selection of markers for self-attribution of humans based on 2 810 SNP genotypes. Symbols show the self-attribution probability p^{sa} that individuals from any of the 7 regions in the data set are correctly attributed to their region. The probability is plotted as a function of the number of SNPs selected at random and with strategies S1 (loci ranked in decreasing within-source diversity), S2 (loci ranked in decreasing between-source diversity) and S3 (reordering the loci ranking of S2 to reduce loci redundancy).

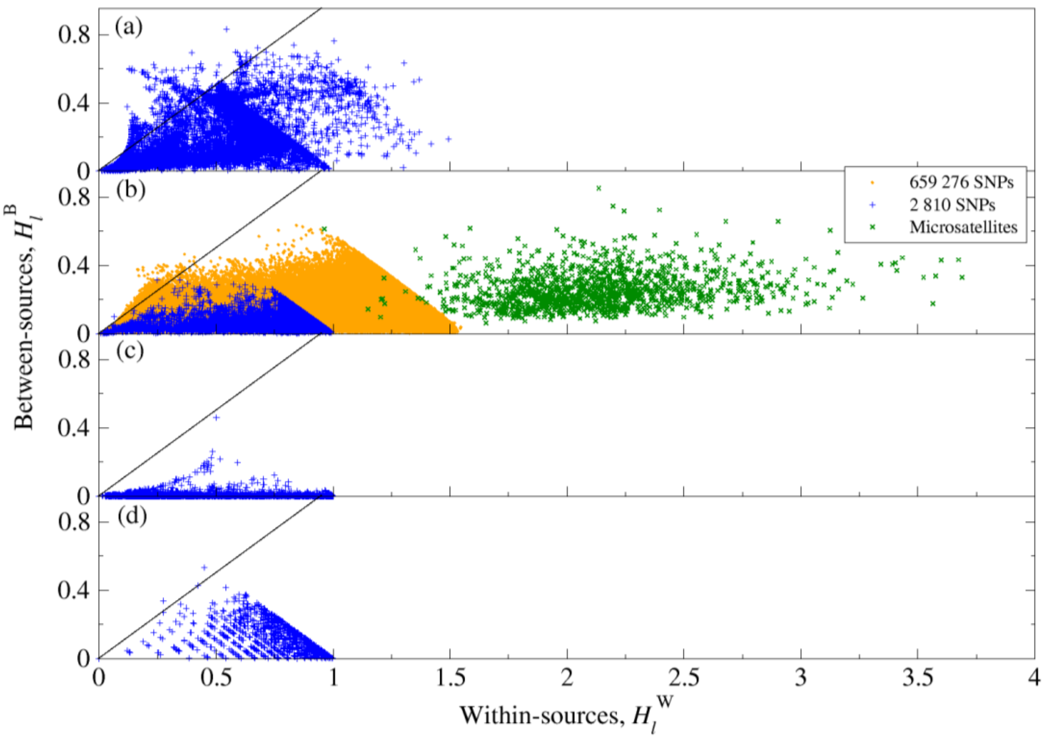

Fig S6. Within- and between-sources allele diversity quantified by entropies. Symbols show the entropy between sources, H_l^B, vs. the entropy within sources, H_l^W, for (a) *Campylobacter* cgSNPs, (b) Human SNPs and microsatellites, (c) *P. californicus* SNPs and (d) breast cancer proteotypes. Most of the data lay on the right of the line $H_l^B = H_l^W$, i.e. the diversity within sources is larger than the entropy between sources for most loci in all the data sets.

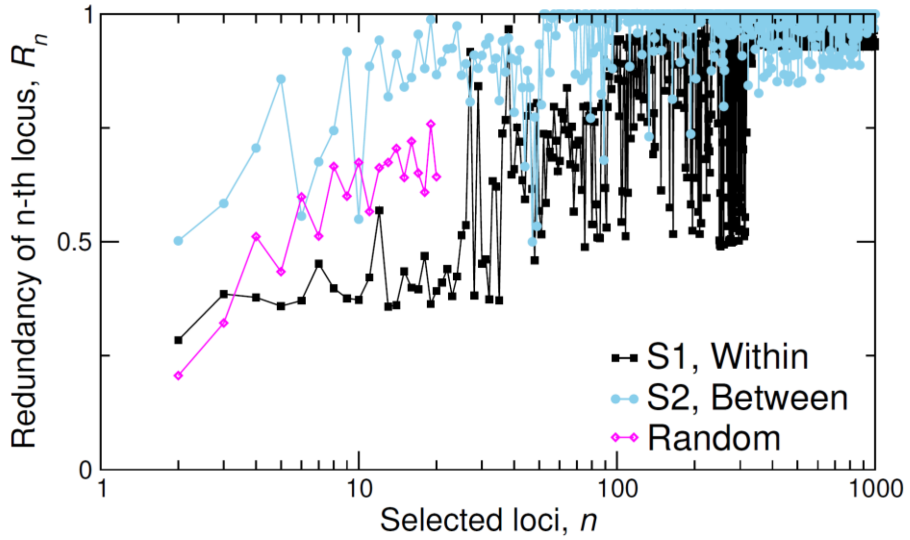

Fig S7. Redundancy R_n of the *n*-th selected locus from 25 938 cgSNP *Campylobacter* genotypes. The redundancy R_n is given by Eq. (6) in the main text. The dependence of R_n on n is shown for randomly selected loci (pink diamonds) and loci ranked with strategies S1 (black squares) and S2 (blue circles).

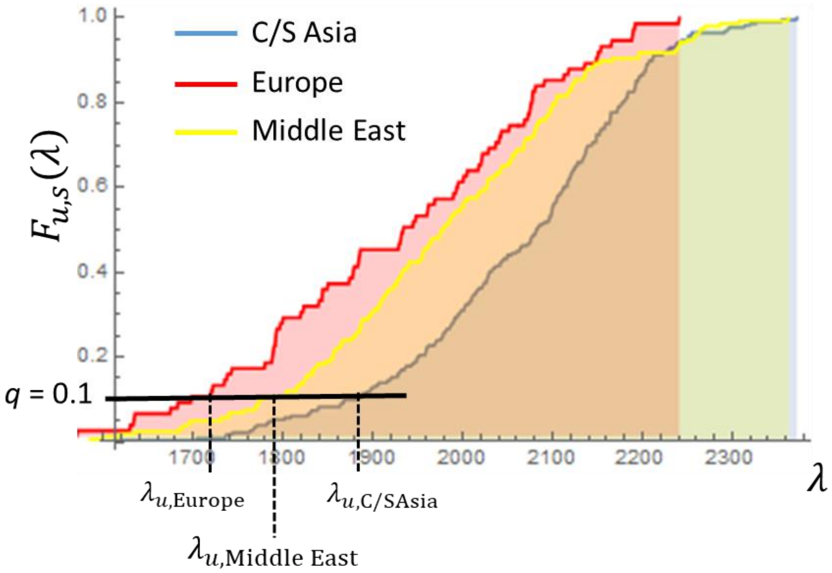

Fig S8. Example of the determination of the minimum *q*-quantile, $\lambda_{min}(q)$, in the MMD method. The curves show the cumulative distribution function $F_{u,s}(\lambda)$ which gives the probability that the Hamming distance between an individual of unknown origin, *u*, and any genotype from source *s* is smaller than λ. This example corresponds to 2810 SNP genotypes of humans from three regions: C/S Asia, Europe and Middle East. For a probability *q* = 0.1, one obtains the minimum *q*-quantile$\lambda_{min}(q) = \lambda_{u,Europe}$. The genotype of *u* is closest to Europe, followed by Middle East and C/S Asia.

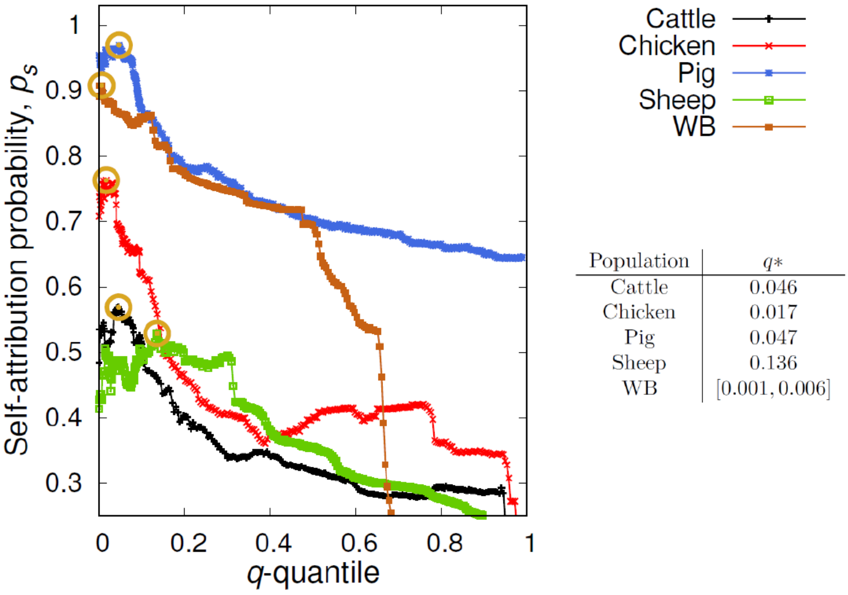

Population	$q*$
Cattle	0.046
Chicken	0.017
Pig	0.047
Sheep	0.136
WB	[0.001, 0.006]

Fig. S9 Self-attribution probability p_s based on 25 938 cgSNP *Campylobacter* genotypes. Different curves show the probability p_s that a removed individual from a food reservoir s (see legend) is correctly attributed to s. The circles indicate the point with maximum self-attribution probabilitiy for each source population. The values q_* (or intervals) of the q-quantile giving the maximum probability are given in the table.

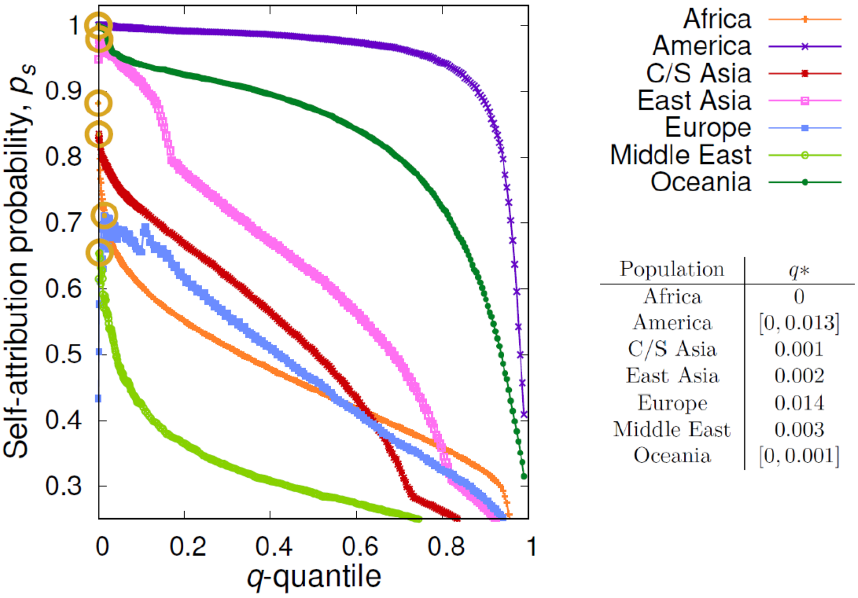

Population	$q*$
Africa	0
America	[0, 0.013]
C/S Asia	0.001
East Asia	0.002
Europe	0.014
Middle East	0.003
Oceania	[0, 0.001]

Fig. S10 Self-attribution probability p_s based on 645 microsatellite human genotypes. Different curves show the probability p_s that a removed individual from region s (see legend) is correctly attributed to s. The circles indicate the point with maximum self-attribution probabilitiy for each source population. The values q_* (or intervals) of the q-quantile giving the maximum probability are given in the table.

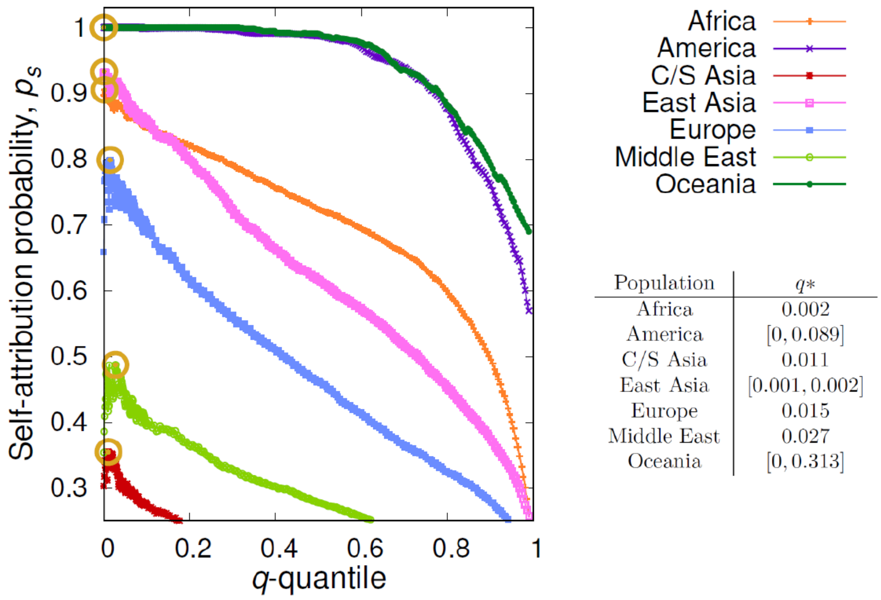

Population	$q*$
Africa	0.002
America	[0, 0.089]
C/S Asia	0.011
East Asia	[0.001, 0.002]
Europe	0.015
Middle East	0.027
Oceania	[0, 0.313]

Fig. S11 Self-attribution probability p_s based on 2 810 SNP human genotypes. Different curves show the probability p_s that a randomly individual from region *s* (see legend) is correctly attributed to *s*. The circles indicate the point with maximum self-attribution probabilitiy for each source population. The values q_* (or intervals) of the q-quantile giving the maximum probability are given in the table.

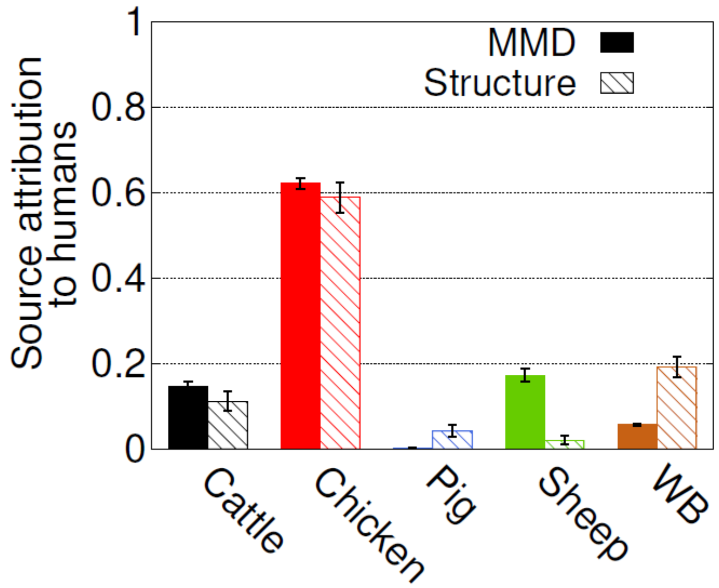

Fig. S12 Source attribution of human 500 human Campylobacter isolates. The bar chart shows the source attribution probability distribution ps obtained with MMD (quantile q=0, solid bars) and STRUCTURE (hatched bars) methods. Results based on 25938 cgSNP genotypes.

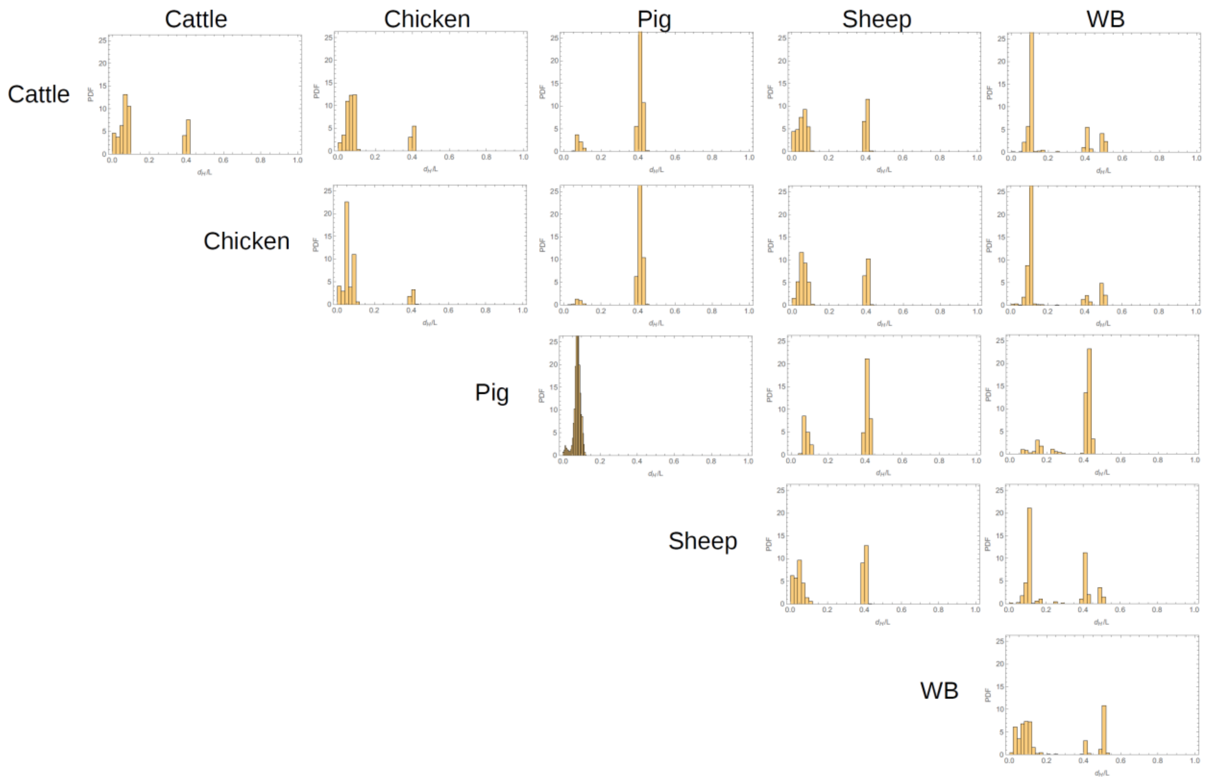

Fig. S13 Hamming distance between and within sources for *Campylobacter* isolates based on 25 938 cgSNP genotypes. Each panel shows the density histogram for the Hamming distance, d_H, between pairs of genotypes from the sources indicated by the row and column labels. The horizontal axis of each plot ranges between 0 and 1 and shows the Hamming distance normalised to the total number of loci, L = 25 938, in each genotype.
