# Supplementary material for: Mining whole genome sequence data to efficiently attribute individuals to source populations
Source: Sci Rep. 2020 Jul 22;10:12124. doi: 10.1038/s41598-020-68740-6 (PMC7376179; doi:10.1038/s41598-020-68740-6)
Supplement: Supplementary file 3 — Supplementary Information 3. [file 41598_2020_68740_MOESM3_ESM.pdf]

Mining Whole Genome Sequence data to efficiently attribute individuals to source populations

Additional file 3: Supplementary tables

Francisco J. Pérez-Reche, Ovidiu Rotariu, Bruno S. Lopes, Ken J. Forbes and Norval J.C. Strachan

Table S1. Allele-frequency divergence for *Campylobacter* sources based on 25 938 cgSNPs. Cells indicate the value of the allele-frequency divergence and blue bars give a visual representation of the value in the cell.

	Cattle	Chicken	Pig	Sheep
WB	0.0645	0.0959	0.3015	0.0828
Sheep	0.0236	0.0583	0.222	
Pig	0.3279	0.4092		
Chicken	0.0292			

Table S2. Allele-frequency divergence for Humans based on 645 microsatellite genotypes. Cells indicate the value of the allele-frequency divergence and blue bars give a visual representation of the value in the cell.

	Africa	America	C/S Asia	East-Asia	Europe	Middle-East
Oceania	0.0412	0.0506	0.0264	0.0248	0.0349	0.033
Middle-East	0.0185	0.0423	0.0079	0.024	0.0044	
Europe	0.0261	0.0412	0.0081	0.0243		
East-Asia	0.0357	0.0316	0.017			
C/S Asia	0.0244	0.035				
America	0.0548					

Table S3. Allele-frequency divergence for Humans based on 2 810 SNP genotypes. Cells indicate the value of the allele-frequency divergence and blue bars give a visual representation of the value in the cell.

	Africa	America	C/S Asia	East-Asia	Europe	Middle-East
Oceania	0.0548	0.0656	0.0404	0.0398	0.0515	0.044
Middle-East	0.0306	0.0438	0.0054	0.0306	0.005	
Europe	0.047	0.0488	0.0073	0.0374		
East-Asia	0.0482	0.0344	0.0239			
C/S Asia	0.0347	0.0377				
America	0.0663					

Table S4. Allele-frequency divergence for Humans based on 659 276 SNP genotypes. Cells indicate the value of the allele-frequency divergence and blue bars give a visual representation of the value in the cell.

	Africa	America	C/S Asia	East-Asia	Europe	Middle-East
Oceania	0.064121636	0.00901646	0.009537095	0.00397227	0.027877831	0.019187964
Middle-East	0.067584773	0.018132307	0.002310077	0.012970799	0.002605231	
Europe	0.088160012	0.025433483	0.00625519	0.020048184		
East-Asia	0.064896711	0.00263804	0.005942497			
C/S Asia	0.064709747	0.010672819				
America	0.068563086					

Table S5. Allele-frequency divergence for breast tumour proteotypes. Cells indicate the value of the allele-frequency divergence and blue bars give a visual representation of the value in the cell.

	ERPR	Her2
TN	0.142210795	0.140692476
Her2	0.126142554	

Table S6. Self-attribution probabilities for *Campylobacter* genotypes. Columns indicate the actual source of isolates used for self-attribution and rows give the inferred source. Numerical values give the probability that genotypes from a source given by a column are attributed to the sources given by the rows. Results are shown for MMD and STRUCTURE (columns marked as "STR").

		Actual source									
		Cattle		Chicken		Pig		Sheep		WB	
		MMD	STR	MMD	STR	MMD	STR	MMD	STR	MMD	STR
Inferred source	Cattle	0.58	0.51	0.06	0.03	0.00	0.00	0.45	0.45	0.00	0.01
	Chicken	0.10	0.34	0.74	0.73	0.05	0.0	0.07	0.23	0.05	0.12
	Pig	0.00	0.09	0.03	0.03	0.95	0.76	0.00	0.18	0.00	0.09
	Sheep	0.31	0.02	0.06	0.02	0.00	0.00	0.45	0.08	0.04	0.00
	WB	0.01	0.04	0.11	0.19	0.00	0.24	0.03	0.06	0.91	0.78

Table S7. Self-attribution probabilities for humans characterised by 645 microsatellite genotypes. Columns indicate the actual source of isolates used for self-attribution and rows give the inferred source. Numerical values give the probability that genotypes from a source given by a column are attributed to the sources given by the rows. Results are shown for MMD and STRUCTURE (columns marked as "STR").

		Actual source													
		Africa		America		C/S Asia		East-Asia		Europe		Middle-East		Oceania	
		MMD	STR	MMD	STR	MMD	STR	MMD	STR	MMD	STR	MMD	STR	MMD	STR
Inferred source	Africa	0.85	0.98	0.00	0.01	0.00	0.01	0.00	0.03	0.00	0.00	0.01	0.06	0.00	0.00
	America	0.00	0.00	1.00	0.87	0.01	0.00	0.02	0.00	0.00	0.00	0.00	0.00	0.00	0.00
	C/S Asia	0.06	0.00	0.00	0.06	0.82	0.93	0.00	0.07	0.02	0.15	0.08	0.05	0.00	0.03
	East-Asia	0.01	0.00	0.00	0.02	0.04	0.01	0.97	0.89	0.01	0.00	0.01	0.00	0.00	0.02
	Europe	0.01	0.00	0.00	0.01	0.08	0.01	0.01	0.01	0.75	0.48	0.23	0.11	0.00	0.00
	Middle-East	0.06	0.02	0.00	0.03	0.05	0.04	0.00	0.01	0.22	0.37	0.67	0.78	0.00	0.01
	Oceania	0.01	0.00	0.00	0.00	0.00	0.00	0.00	0.00	0.00	0.00	0.00	0.00	1.0	0.94

Table S8. Self-attribution probabilities for humans characterised by 2 810 SNP genotypes. Columns indicate the actual source of isolates used for self-attribution and rows give the inferred source. Numerical values give the probability that genotypes from a source given by a column are attributed to the sources given by the rows. Results are shown for MMD and STRUCTURE (columns marked as "STR").

		Actual source													
		Africa		America		C/S Asia		East-Asia		Europe		Middle-East		Oceania	
		MMD	STR	MMD	STR	MMD	STR	MMD	STR	MMD	STR	MMD	STR	MMD	STR
Inferred source	Africa	0.91	0.96	0.00	0.00	0.00	0.00	0.00	0.00	0.00	0.00	0.02	0.04	0.00	0.00
	America	0.00	0.00	1.00	1.00	0.04	0.00	0.06	0.00	0.01	0.00	0.00	0.00	0.00	0.00
	C/S Asia	0.00	0.00	0.00	0.00	0.36	0.87	0.00	0.00	0.05	0.03	0.04	0.06	0.00	0.00
	East-Asia	0.01	0.00	0.00	0.00	0.11	0.01	0.93	1.00	0.01	0.00	0.01	0.00	0.00	0.00
	Europe	0.03	0.00	0.00	0.00	0.35	0.05	0.00	0.00	0.80	0.80	0.38	0.17	0.00	0.00
	Middle-East	0.05	0.04	0.00	0.00	0.11	0.07	0.00	0.00	0.13	0.17	0.54	0.73	0.00	0.00
	Oceania	0.00	0.00	0.00	0.00	0.03	0.00	0.01	0.00	0.00	0.00	0.01	0.00	1.00	1.00

Table S9. Self-attribution probabilities for humans characterised by 659 276 SNP genotypes. Columns indicate the actual source of isolates used for self-attribution and rows give the inferred source. Numerical values give the probability that genotypes from a source given by a column are attributed to the sources given by the rows. Results are shown for MMD and ADMIXTURE (columns marked as "ADM").

		Actual source													
		Africa		America		C/S Asia		East-Asia		Europe		Middle-East		Oceania	
		MMD	ADM	MMD	ADM	MMD	ADM	MMD	ADM	MMD	ADM	MMD	ADM	MMD	ADM
Inferred source	Africa	1.00	0.97	0.00	0.00	0.00	0.01	0.00	0.00	0.00	0.00	0.00	0.03	0.00	0.00
	America	0.00	0.00	1.00	0.95	0.00	0.00	0.00	0.01	0.00	0.01	0.00	0.00	0.00	0.00
	C/S Asia	0.00	0.00	0.00	0.00	0.90	0.89	0.00	0.05	0.00	0.06	0.00	0.05	0.00	0.00
	East-Asia	0.00	0.00	0.00	0.02	0.10	0.02	1.00	0.91	0.00	0.01	0.00	0.00	0.00	0.07
	Europe	0.00	0.00	0.00	0.02	0.00	0.02	0.00	0.03	1.00	0.87	0.10	0.11	0.00	0.00
	Middle-East	0.00	0.03	0.00	0.01	0.00	0.04	0.00	0.00	0.00	0.05	0.90	0.81	0.00	0.00
	Oceania	0.00	0.00	0.00	0.00	0.00	0.00	0.00	0.00	0.00	0.00	0.00	0.00	1.00	0.93
