# Supplementary material for: Mining whole genome sequence data to efficiently attribute individuals to source populations
Source: Sci Rep. 2020 Jul 22;10:12124. doi: 10.1038/s41598-020-68740-6 (PMC7376179; doi:10.1038/s41598-020-68740-6)
Supplement: Supplementary file 4 — Supplementary Information 4. [file 41598_2020_68740_MOESM4_ESM.pdf]

Mining Whole Genome Sequence data to efficiently attribute individuals to source populations

Additional file 4: Giant California sea cucumber (*Parastichopus californicus*) example

Francisco J. Pérez-Reche, Ovidiu Rotariu, Bruno S. Lopes, Ken J. Forbes and Norval J.C. Strachan

The dataset used for assignment of the giant California sea cucumber comprised 717 individuals from the northeastern Pacific Ocean coast. The genotype of each individual was described by 3 699 SNPs [1]. The format of the data file used by the MMD method is described in Additional file 1: Supplementary data file S5.

In Ref. [1], a leave-one out strategy [2] was used for self-attribution of *P. californicus* to the north and south regions of the coast. The leave-one out strategy consists in removing an individual from the dataset which is attributed to the sources that are described by the remaining genotypes. As for the rest of examples studied in this work, we used a Monte-Carlo crossvalidation strategy [2] for self-attribution with the MMD method. In particular, we removed pairs of individuals (i.e. $I_u = 2$) whose origin was assumed to be unknown. This procedure was repeated 100 times by randomly removing pairs of individuals from each region.

The probability of correct self-attribution $p^{\rm sa}$ for a removed pair was estimated for each realisation. Individuals from the north were correctly attributed with probability $p^{\rm sa} = 1$ in most of the realisations (see the histogram for $p^{\rm sa}$ in Fig. AF4.1(a)). On average, the self-attribution accuracy for the north region was 92%. This is close to the 90% accuracy reported for the leave-one-out method in [1]. The probability of correct self-attribution for individuals from the south region is more widely spread than that for the north. It ranges between ~ 0.6 and 1 (Fig. AF4.1(b)) which is statistically compatible with the 88% correct self-attribution reported in Ref. [1]. The mean self-attribution accuracy in this case is 71%.

With regards to the selection of informative *P. californicus* loci, self-attribution is slightly more accurate with strategy S1 but differences are not significant for selections of more than 100 SNPs (Fig. AF4.2). An overall self-attribution accuracy of 76% was achieved by selecting the 100 most informative loci with strategy S1 (this accuracy is only $\sim 8\%$ lower than that obtained with all loci). This trend is statistically compatible with the findings in [1] which reported a self-attribution success of $\sim 80\%$ when selecting 100 SNPs.

[1] A. Xuereb, L. Benestan, É. Normandeau, R. M. Daigle, J. M. R. Curtis, L. Bernatchez, and M.-J. Fortin, Molecular Ecology **27**, 2347 (2018).

[2] M. Kuhn and K. Johnson, *Applied Predictive Modeling* (Springer New York, New York, NY, 2013).

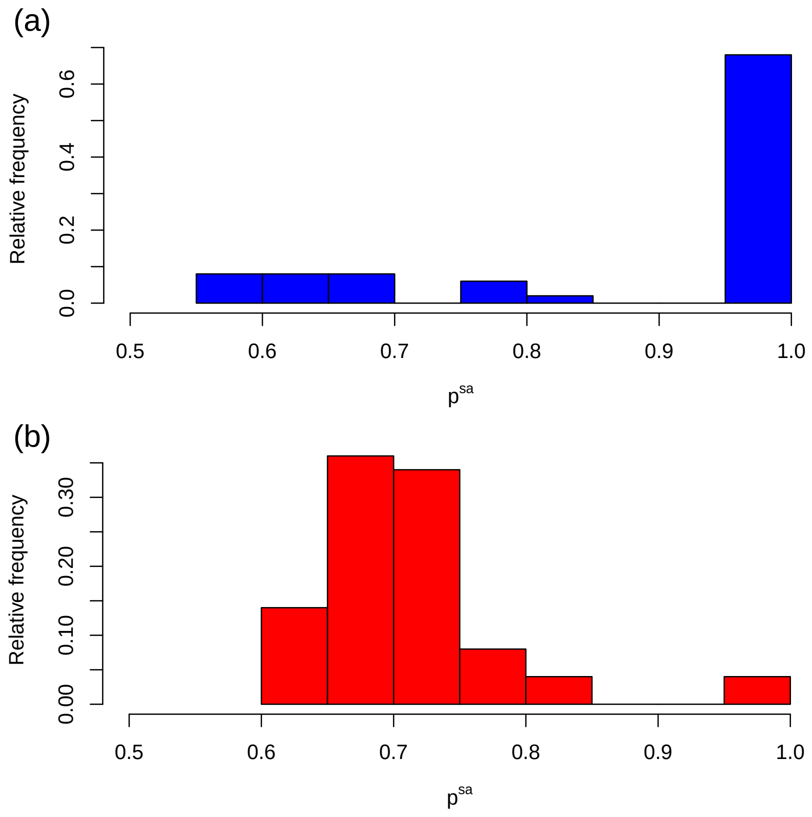

Fig. AF4.1. **Correct self-attribution of *P. californicus*.** Histograms show the relative frequency of the probability of correct self-attribution, p^{sa}, of samples from (a) north and (b) south regions obtained by randomly removing pairs of individuals from each region.

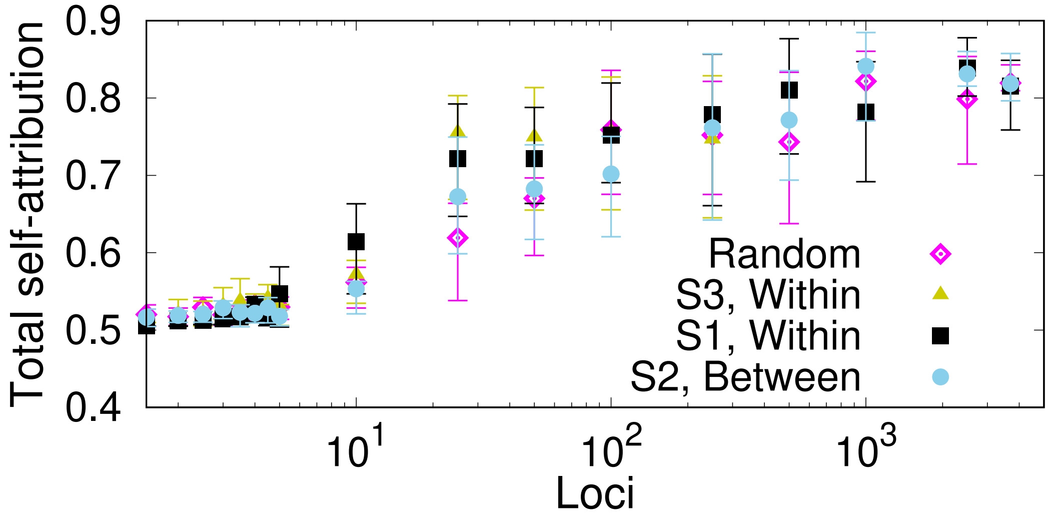

Fig. AF4.2. **Selection of markers for self-attribution of *P. californicus* marine cucumber genotypes.** Symbols show the probability of correct self-attribution, p^{sa}, for samples from the north and south regions of the northeastern Pacific Ocean North American coast. The probability is plotted as a function of the number of SNPs selected at random and with strategies S1 (loci ranked in decreasing within-source diversity), S2 (loci ranked in decreasing between-source diversity) and S3 (reordering the loci ranking of S1 to reduce loci redundancy). Results were obtained by removing pairs of samples from sources to be attributed as if their origin was unknown. The process was repeated 100 times for each selected pair.
