# Supplementary material for: Mining whole genome sequence data to efficiently attribute individuals to source populations
Source: Sci Rep. 2020 Jul 22;10:12124. doi: 10.1038/s41598-020-68740-6 (PMC7376179; doi:10.1038/s41598-020-68740-6)
Supplement: Supplementary file 5 — Supplementary Information 5. [file 41598_2020_68740_MOESM5_ESM.pdf]

Mining Whole Genome Sequence data to efficiently attribute individuals to source populations

Additional file 5: Breast cancer proteomic example

Francisco J. Pérez-Reche, Ovidiu Rotariu, Bruno S. Lopes, Ken J. Forbes and Norval J.C. Strachan

The dataset used in the breast cancer example comprised 40 breast cancer samples of three subtypes: 14 oestrogen receptor and/or progesterone receptor positive (ERPR positive) cases, 15 epidermal growth factor receptor *ErbB2/Her2* positive (Her2 positive) cases and 11 triple negative (TN) cases [1]. For each sample, the data from Ref. [1] provide the mass spectrum intensity I_{MS} detected at 65 533 discrete values of m/z (ionic mass per unit charge). In order to represent these data as a feature vector suitable for MMD, the mass spectrum for each sample was transformed by replacing positive values of I_{MS} by 1. This resulted in a feature vector of 65 533 elements with values 0 or 1 which defines a proteotype for each sample (Additional file 1: Suppl data file S6). The feature vector for each sample defines a multilocus proteotype analogous to the multilocus genotypes used in the *Campylobater*, human and *P. californicus* examples.

Self-attribution was performed by a Monte-Carlo cross-validation strategy [2] similar to that used for *P. californicus*, i.e. $I_u = 2$ samples were randomly removed whose cancer subtypes were assumed to be unknown. This procedure is repeated for 100 different selections of pairs. Since the number of samples in the proteomic dataset is relatively small (40 samples), removing few samples is important to make sure that the remaining samples represent the sources as accurately as possible.

Overall, cancer samples were correctly attributed to their subtype (ERPR, Her2 or TN) in 63% of the cases. The average self-attribution probabilities for ERPR, Her2 and TN tumours were 0.64, 0.57 and 0.68, respectively (see Fig. AF5.1). Wrong self-attribution of any of the subtypes was approximately evenly distributed among the two wrong subtypes.

Self-attribution of breast cancer tumours is not significantly affected by the strategy used to select loci (Fig. AF5.2). Attribution accuracy saturates for selections of more than ~ 500 loci irrespective of the strategy used for loci selection.

[1] S. Tyanova, R. Albrechtsen, P. Kronqvist, J. Cox, M. Mann, and T. Geiger, Nature Communications **7**, 1 (2016).

[2] M. Kuhn and K. Johnson, *Applied Predictive Modeling* (Springer New York, New York, NY, 2013).

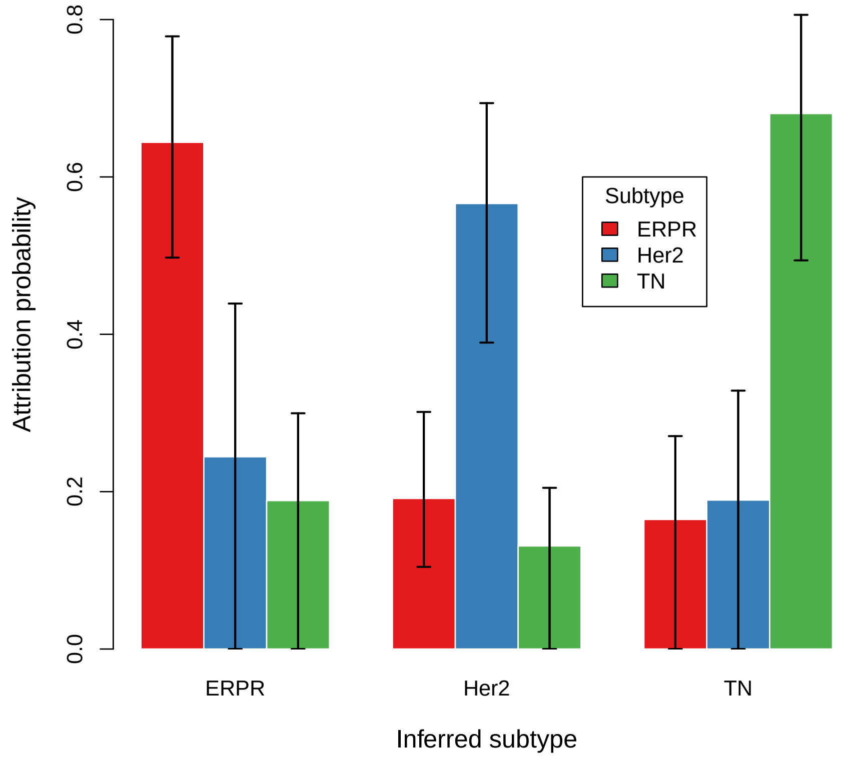

Fig. AF5.1. **Self-attribution of breast cancer tumours based on proteomic data.** Each sample (40 in total) is described by a 65 533 loci proteotype. Different colours, indicated in the legend, correspond to different cancer subtypes (ERPR, Her2 and TN). The bars for a given subtype provide the probability $p_{u,s}$ that removed samples, u, from this subtype are attributed to each of the possible sources, s. The probability indicated by the bars corresponds to the mean assignment probability over different selections. On average, ERPR, Her2 and TN subtypes are correctly attributed in 64%, 57% and 68% of the cases, respectively.

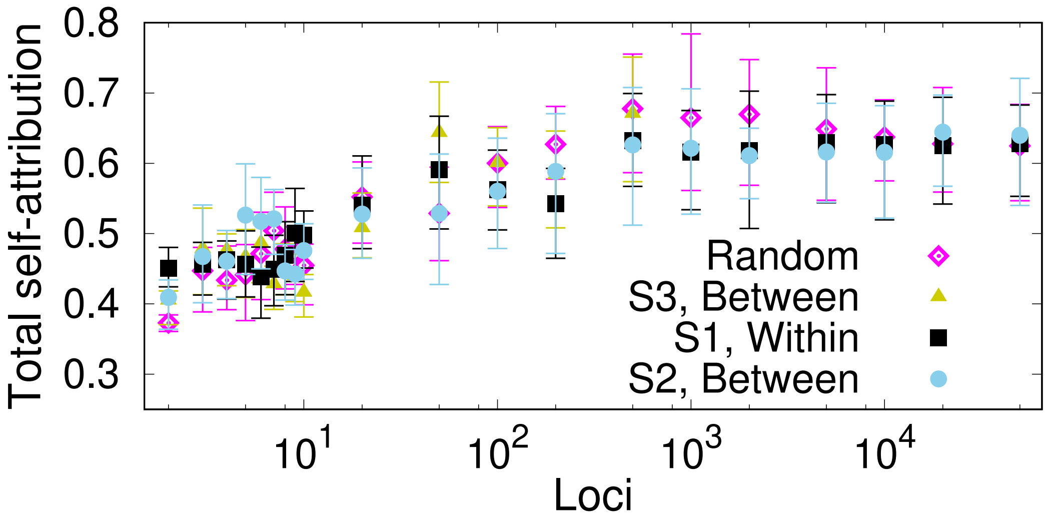

Fig. AF5.2. **Selection of markers for self-attribution of breast cancer proteotypes.** Symbols show the self-attribution probability p^{sa} that individuals from any of the three cancer subtypes (ERPR, Her2 or TN) are correctly attributed to their source. The probability is plotted as a function of the number of SNPs selected at random and with strategies S1 (loci ranked in decreasing within-source diversity), S2 (loci ranked in decreasing between-source diversity) and S3 (reordering the loci ranking of S2 to reduce loci redundancy).
