# Supplementary material for: Mining whole genome sequence data to efficiently attribute individuals to source populations
Source: Sci Rep. 2020 Jul 22;10:12124. doi: 10.1038/s41598-020-68740-6 (PMC7376179; doi:10.1038/s41598-020-68740-6)
Supplement: Supplementary file 6 — Supplementary Information 6. [file 41598_2020_68740_MOESM6_ESM.pdf]

Mining Whole Genome Sequence data to efficiently attribute individuals to source populations

Additional file 6: Comparison of the MMD with other methods - Analytical considerations

Francisco J. Pérez-Reche, Ovidiu Rotariu, Bruno S. Lopes, Ken J. Forbes and Norval J.C. Strachan

I. MMD METHOD IN TERMS OF ALLELE PROBABILITIES

Here, we present a description of the MMD method in terms of allele probabilities which is useful to compare with assignment methods that rely on allele probabilities. Our description applies to the particular case in which genotypes consist of L *unlinked* loci with two alleles each. Under this assumption, the Hamming distance $d_{\mathrm{H}}(\mathbf{u}, \mathbf{a}_{i,s})$ is a random variable obeying a Poisson's Binomial distribution [1] with success probabilities $\{1 - \pi_{u_l,l,s}\}_{l=1}^{L}$. Here, $\pi_{u_l,l,s}$ is the probability that the allele u_l in the individual to be assigned is observed at locus l in source s. In general, $\pi_{a,l,s}$ denotes the probability of allele a at locus l in population s.

The measures of similarity between individuals and sources used in previous work based on allele frequencies can be viewed as *particular characteristics of the Hamming distance distribution* used by the MMD method. For instance, the likelihood function,

$$\mathcal{L}_{u,s} = \prod_{l=1}^{L} \pi_{u_l,l,s} \ , \tag{1}$$

used in many assignment tests [2–7] corresponds to the probability that $d_{\mathrm{H}}(\mathbf{u}, \mathbf{a}_{i,s}) = 0$, i.e. the probability that the genotype $\mathbf{u}$ exists in source s. Genetic distances used in distance-based assignment tests [4, 5], can also be expressed in terms of the probabilities $\{\pi_{u_l,l,s}\}$. For example, Nei's D_A distance [8] between the individual to be assigned and source s is

$$D_A = 1 - L^{-1} \sum_{l=1}^{L} \pi_{u_l,l,s} \ .$$

We note that some classical genetic distances [8] such as Nei's standard genetic distance, D_S, or Nei's minimum genetic distance, D_m, depend on the gene identity [9] of the sources, $J_s = L^{-1} \sum_{l=1}^{L} \sum_{a \in \mathcal{A}} \pi_{a,l,s}$, in addition to the probabilities $\{\pi_{u_l,l,s}\}$. For example, Nei's standard genetic distance between $\mathbf{u}$ and source s is

$$D_S = -\ln \left[\frac{\sum_{l=1}^{L} \pi_{u_l,l,s}}{L\sqrt{J_s}} \right]$$

The gene identity is intrinsic to sources and does not reflect the similarity between the individual to be attributed and sources. In general, methods based on D_S and D_m will predict a higher attribution to the source with lower gene identity but this has nothing to do with the individual to be attributed.

II. ATTRIBUTION ERRORS ASSOCIATED WITH ERRORS IN ALLELE PROBABILITIES

As mentioned in the main text, errors in the estimates of allele probabilities $\{\pi_{a,l,s}\}$ used to characterise sources will induce an error in attribution. Here we estimate the dependence of the attribution error on the number L of loci in the genotypes and the number I_s of genotypes used to describe each source.

A. Attribution error for the MMD method

For the MMD method, errors in the estimates of the allele probabilities propagate to the quantile $\lambda_{u,s}(q)$, score $\sigma_{u,s}$ and attribution probability $p_{u,s}$ defined in the Methods of the main text. The dependence of the errors of $\lambda_{u,s}(q)$ and $\sigma_{u,s}$ on L and I_s can be estimated for a simple model for unlinked loci in which alleles have the same probability distribution for all loci, i.e. a model with $\pi_{u_l,l,s} = r_s$ independently of l. In this case, the Hamming distance obeys a binomial distribution for L Bernoulli trials with probability of success $1 - r_s$. In the limit of large L, the binomial distribution can be approximated by a normal distribution with mean $\mu_s = L(1 - r_s)$ and variance $\Delta_s^2 = Lr_s(1 - r_s)$. Under these assumptions, the quantile $\lambda_{u,s}(q)$ satisfies

$$\lambda_{u,s}(q) = \mu + \Phi^{-1}(q)\Delta_s \ , \tag{2}$$

and the score $\sigma_{u,s}$ quantifying the proximity of genotype $\mathbf{u}$ to source s is

$$\sigma_{u,s} = \Phi\left(\frac{\lambda_{\text{min}} - \mu_s}{\Delta_s}\right) \ . \tag{3}$$

Here, $\lambda_{\text{min}} = \min_s\{\lambda_{u,s}(q)\}$ and $\Phi^{-1}(x)$ is the inverse of the cumulative distribution function for the standard normal distribution.

From Eq. (2), the error of $\lambda_{u,s}(q)$ in the limit of extended genotypes with large L is given by

$$\delta\lambda_{u,s} = \left|\frac{\partial\lambda_{u,s}}{\partial r_s}\right| \delta r_s \simeq L\delta r_s \ . \tag{4}$$

Here, δr_s is the error in the allele probabilities. In the MMD method and other methods that approximate these probabilities by the observed allele frequencies, the error is $\delta r_s = O(I_s^{-1/2})$. Therefore,

$$\delta\lambda_{u,s} \simeq LI_s^{-1/2} \ . \tag{5}$$

Since $\lambda_{u,s} \simeq L$ (cf. Eq. (2)), we conclude that the relative error of $\lambda_{u,s}$ is $\delta\lambda_{u,s}/\lambda_{u,s} = O(I_s^{-1/2})$, i.e. it does not increase with the number of loci, L.

Let us denote the closest source to individual $\mathbf{u}$ as s_{closest} (this is the source with $\lambda_{u,s_{\text{closest}}} = \lambda_{\text{min}}$). From Eq. (3), the error in the assignment score $\sigma_{u,s}$ is given by:

$$\delta\sigma_{u,s} = \left|\frac{\partial\sigma_{u,s}}{\partial r_s}\right| \delta r_s + \left|\frac{\partial\sigma_{u,s}}{\partial\lambda_{\text{min}}}\right| \delta\lambda_{\text{min}} \simeq \begin{cases} aL^{1/2}e^{-bL^2}\delta r_s \ , & \text{for } s \neq s_{\text{closest}} \\ a'L^{1/2}\delta r_s \ , & \text{for } s = s_{\text{closest}} \ . \end{cases} \tag{6}$$

Here, a, a' and b are independent of L and we have assumed that δr_s is approximately the same for all sources, including s_{closest}. One can show that the error for the attribution probability $p_{u,s}$ is proportional to that of $\delta\sigma_{u,s}$.

To summarise, our arguments show that the assignment error for the MMD method is $O(L^{1/2})$. In the particular case in which the allele probabilities are estimated by frequencies, one has $\delta r_s = O(I_s^{-1/2})$ and the assignment error is $O(L^{1/2}I_s^{-1/2})$, i.e. it increases with L and decreases with the number of genotypes used to define the sources.

B. Attribution error for the likelihood function

The error for the likelihood function given by Eq. (1) can be easily calculated as a function of the errors $\{\delta\pi_{u_l,l,s}\}$ for the allele probabilities. Propagation of errors gives

$$\delta\mathcal{L}_{u,s} = \sum_{l=1}^{L} \left| \frac{\partial \mathcal{L}_{u,s}}{\partial \pi_{u_l,l,s}} \right| \delta\pi_{u_l,l,s} = \mathcal{L}_{u,s} \sum_{l=1}^{L} \frac{\delta\pi_{u_l,l,s}}{\pi_{u_l,l,s}} \simeq L\mathcal{L}_{u,s} \ , \tag{7}$$

where we have assumed $\delta\pi_{u_l,l,s} > 0$ for all loci.

According to Eq. (7), the relative error of the likelihood function, $\delta\mathcal{L}_{u,s}/\mathcal{L}_{u,s}$, increases with L unless the errors in the probability estimates, $\{\delta\pi_{u_l,l,s}\}$, are zero.

The log-likelihood function is more commonly used than the likelihood itself. One can easily show that the error for the log-likelihood function typically equals the relative error of $\mathcal{L}_{u,s}$ and is therefore $O(L)$. This shows that attribution errors based on a likelihood function increase faster with L than those for the MMD method.

[1] Y. H. Wang, Statistica Sinica **3**, 295 (1993).
[2] B. Rannala and J. L. Mountain, Proceedings of the National Academy of Sciences **94** (1997).
[3] D. Paetkau, W. Calvert, I. Stirling, and C. Strobeck, Molecular Ecology **4**, 347 (1995).
[4] J. M. Cornuet, S. Piry, G. Luikart, A. Estoup, and M. Solignac, Genetics **153**, 1989 (1999).
[5] S. Piry, A. Alapetite, J.-M. Cornuet, D. Paetkau, L. Baudouin, and A. Estoup, Journal of Heredity **95**, 536 (2004).
[6] L. Mughini-Gras and W. van Pelt, International Journal of Food Microbiology **191**, 109 (2014).
[7] J. K. Pritchard, M. M. Stephens, and P. Donnelly, Genetics **155**, 945 (2000).
[8] N. Takezaki and M. Nei, Genetics **144**, 389 (1996).
[9] M. Nei, Proceedings of the National Academy of Sciences **70**, 3321 (1973).
