# Supplementary material for: Mining whole genome sequence data to efficiently attribute individuals to source populations
Source: Sci Rep. 2020 Jul 22;10:12124. doi: 10.1038/s41598-020-68740-6 (PMC7376179; doi:10.1038/s41598-020-68740-6)
Supplement: Supplementary file 7 — Supplementary Information 7. [file 41598_2020_68740_MOESM7_ESM.pdf]

Mining Whole Genome Sequence data to efficiently attribute individuals to source populations

Additional file 7: Exclusion method

Francisco J. Pérez-Reche, Ovidiu Rotariu, Bruno S. Lopes, Ken J. Forbes and Norval J.C. Strachan

Here, we apply the threshold exclusion method proposed in [1] to the MMD source attribution results. The exclusion method consists in setting a threshold T for the probability $p_{u,s}$ such that an individual u is assigned to source s if $p_{u,s} \geq T$. Otherwise, if $p_{u,s} < T$, the individual cannot be attributed to the source s. When $p_{u,s} < T$ for all the sampled sources, $s \in \mathcal{S}$, the individual is not assigned to any source. The performance of this method was explored to test the MMD attribution results for human genotypes with 659 276 SNPs and *Campylobacter* genotypes with 25 937 SNPs. For the human example, we focused on self-attribution to 7 geographical regions. In this case, a very small proportion of genotypes are excluded even for very selective values of T (see Fig. AF7.1). In fact, only 2% of individuals are excluded from all regions for $T = 1$. In fact, for $T = 1$, all individuals are attributed to the correct source except for 10% of individuals that are excluded from Middle East and 1% that are excluded from C/S Asia. To some extent, the high accuracy found for this dataset could be expected since we are dealing with self-attribution and the true region of individuals has been sampled for sure.

Exclusion is more prominent for *Campylobacter* isolates (see Fig. AF7.2). For $T > 0.9$, more than 60% of isolates from human patients are excluded from all sources. Since the origin of human isolates is unknown, one could conclude that there is a high percentage of isolates that originated from sources that were not sampled. However, this is not a solid conclusion since the method also predicts high exclusion percentages (> 37% on average for $T > 0.9$) for isolates from food and animal sources whose true source is known. The exclusion percentage is particularly high for sheep (all isolates excluded from all sources for $T > 0.7$) and cattle (52% excluded for any $T > 0.8$). High exclusion rates in this example are likely due to a low genetic differentiation between sources. In this situation, forcing assignment to a single source is not well justified.

---

[1] S. Manel, P. Berthier, and G. Luikart, Conservation Biology **16**, 650 (2002).

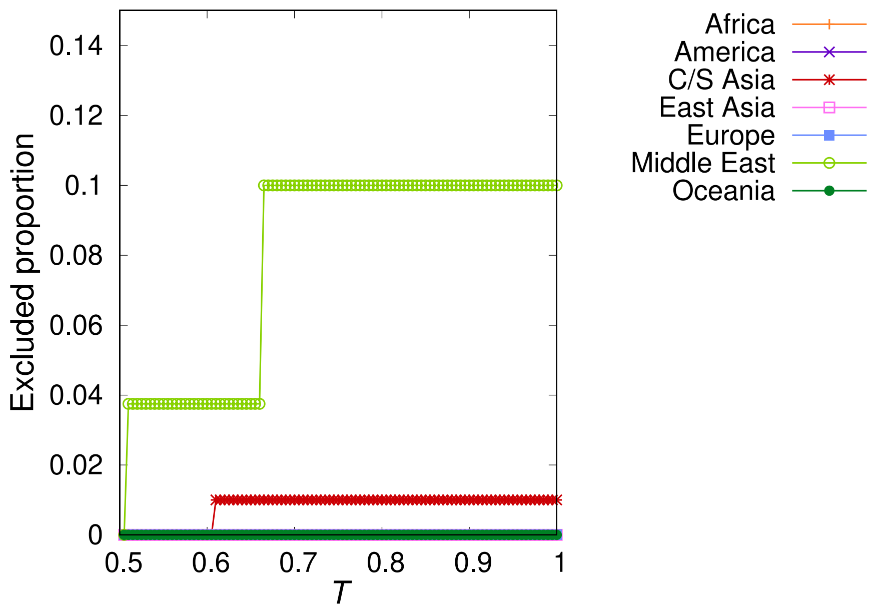

Fig. AF7.1. **Exclusion test for humans based on 659 276 SNP genotypes.** For a given geographical region, the proportion of individuals that are not attributed to any region (i.e. individuals with $p_{u,s} < T$ for all regions, s) is plotted as a function of the exclusion threshold, T. Different symbols correspond to individuals from different regions, as marked by the legend.

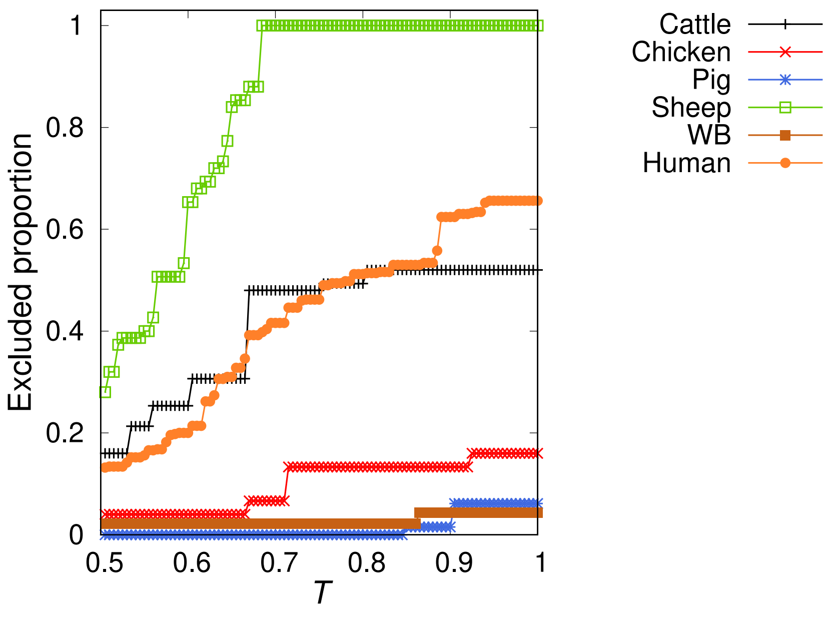

Fig. AF7.2. **Exclusion test for Campylobacter isolates based on 25 938 cgSNP genotypes.** For a given Campylobacter reservoir, the proportion of isolates that are not attributed to any source (i.e. isolates with $p_{u,s} < T$ for all sources) is plotted as a function of the exclusion threshold, T. Different symbols correspond to isolates from different reservoirs, as marked by the legend.
